# Resource Allocation During the Transition to Diazotrophy in *Klebsiella oxytoca*

**DOI:** 10.3389/fmicb.2021.718487

**Published:** 2021-08-09

**Authors:** Christopher J. Waite, Anya Lindström Battle, Mark H. Bennett, Matthew R. Carey, Chun K. Hong, Ioly Kotta-Loizou, Martin Buck, Jörg Schumacher

**Affiliations:** Department of Life Sciences, Imperial College London, London, United Kingdom

**Keywords:** nitrogen stress, absolute protein quantification, *Klebsiella oxytoca*, *nif* gene expression, PII proteins, nitrogenase, diazotrophic bacteria: biological fixation of nitrogen, resource allocation

## Abstract

Free-living nitrogen-fixing bacteria can improve growth yields of some non-leguminous plants and, if enhanced through bioengineering approaches, have the potential to address major nutrient imbalances in global crop production by supplementing inorganic nitrogen fertilisers. However, nitrogen fixation is a highly resource-costly adaptation and is de-repressed only in environments in which sources of reduced nitrogen are scarce. Here we investigate nitrogen fixation (*nif*) gene expression and nitrogen starvation response signaling in the model diazotroph *Klebsiella oxytoca* (*Ko*) M5a1 during ammonium depletion and the transition to growth on atmospheric N_2_. Exploratory RNA-sequencing revealed that over 50% of genes were differentially expressed under diazotrophic conditions, among which the *nif* genes are among the most highly expressed and highly upregulated. Isotopically labelled QconCAT standards were designed for multiplexed, absolute quantification of Nif and nitrogen-stress proteins *via* multiple reaction monitoring mass spectrometry (MRM-MS). Time-resolved Nif protein concentrations were indicative of bifurcation in the accumulation rates of nitrogenase subunits (NifHDK) and accessory proteins. We estimate that the nitrogenase may account for more than 40% of cell protein during diazotrophic growth and occupy approximately half the active ribosome complement. The concentrations of free amino acids in nitrogen-starved cells were insufficient to support the observed rates of Nif protein expression. Total Nif protein accumulation was reduced 10-fold when the NifK protein was truncated and nitrogenase catalysis lost (*nifK*_1__–__1__203_), implying that reinvestment of *de novo* fixed nitrogen is essential for further *nif* expression and a complete diazotrophy transition. Several amino acids accumulated in non-fixing Δ*nifLA* and *nifK*_1__–__1203_ mutants, while the rest remained highly stable despite prolonged N starvation. Monitoring post-translational uridylylation of the PII-type signaling proteins GlnB and GlnK revealed distinct nitrogen regulatory roles in *Ko* M5a1. GlnK uridylylation was persistent throughout the diazotrophy transition while a Δ*glnK* mutant exhibited significantly reduced Nif expression and nitrogen fixation activity. Altogether, these findings highlight quantitatively the scale of resource allocation required to enable the nitrogen fixation adaptation to take place once underlying signaling processes are fulfilled. Our work also provides an omics-level framework with which to model nitrogen fixation in free-living diazotrophs and inform rational engineering strategies.

## Introduction

Most organisms are unable to directly assimilate inert atmospheric dinitrogen (N_2_) and rely instead on the bio-availability of chemically reduced, or “fixed,” forms of nitrogen (N), such as ammonia, nitrate or amino acids. Food security is fundamentally dependent on the uptake of fixed N by agricultural crops. Biological N fixation – the reduction of N_2_ to NH_3_ by specialised microorganisms – replenishes most of the fixed N in the biosphere and supports approximately 60% of the N required for human nutrition (Smil, 2001). However, as crop production must increase by up to 70% to support the estimated global food demand in 2050 (Hunter et al., 2017), agriculture is growing ever more reliant on the synthetically fixed and environmentally damaging N fertilisers produced by the industrial Haber–Bosch process. Addressing the severe anthropogenic imbalances in the global N cycle is critical to managing the triple global challenges of food security, climate change, and environmental degradation (Steffen et al., 2015; Zhang et al., 2015). Modern biotechnological approaches offer great promise for expanding the capacity of biological N fixation and the development of more sustainable solutions for the delivery of N to plants (e.g., Rogers and Oldroyd, 2014; Batista and Dixon, 2019).

Biological N fixation, widely distributed among bacterial lineages and compatible with a range of physiologies, is catalysed by the structurally and mechanistically conserved nitrogenase enzyme (Dixon and Kahn, 2004). The universal molybdenum-iron (MoFe) nitrogenase consists of two oxygen-sensitive metalloproteins. The MoFe protein is a heterotetramer of NifD and NifK subunits and contains an iron-molybdenum cofactor in each catalytic site for N_2_ reduction. The iron (Fe) protein, which acts as an electron donor for MoFe, is a homodimer of NifH. N_2_ reduction occurs with a slow catalytic turnover rate (∼5 s^–1^) (Hageman and Burris, 1978) and an optimal stoichiometry of: N_2_ + 8 H^+^ + 8 e^–^ + 16 MgATP → 2 NH_3_ + H_2_ + 6 MgADP + 16 P_*i*_ (Thorneley and Lowe, 1985; Seefeldt et al., 2009). As such, diazotrophic growth on N_2_ as a sole N source necessitates both a high nitrogenase concentration (up to 20% of total cell protein) and considerable energetic (ATP) input (Dixon and Kahn, 2004).

The 24-kb nitrogen fixation (*nif*) gene cluster of the free-living diazotroph *Klebsiella oxytoca* (*Ko*) has been the model system for molecular biology studies of biological nitrogen fixation (Arnold et al., 1988). Consisting of 20 genes arranged into 8 operons, this cluster is sufficient for heterologous N fixation in non-diazotrophs such as *Escherichia coli* (Dixon and Postgate, 1972). Besides the structural proteins NifHDK, many other *nif* gene products function coordinately in the assembly of the mature nitrogenase complex: synthesis and insertion of the FeMo-cofactor (NifBEQNVXY); synthesis of Fe–S and P metalloclusters (NifSUZ); maturation of MoFe protein (NifWYZ); maturation of (NifMW); and electron transport to (NifFJ) the Fe protein (Jimenez-Vicente et al., 2018; Mus et al., 2018). The products of the *nifLA* operon regulate the transcription of the other *nif* operons *via* conserved promoter elements. Given the significant energetic costs associated with nitrogenase expression and function, diazotrophs utilise common regulatory principles to restrict *nif* gene expression to the optimal environmental conditions. Among proteobacteria, the σ^54^-type bacterial enhancer binding protein (bEBP) NifA stringently couples *nif* transcription to the availability of fixed N, energy status, and microaerobic oxygen concentration (Dixon and Kahn, 2004). In *Ko*, NifA is sequestered in an inactive form in non-optimal conditions by the anti-activator flavoprotein NifL (Henderson et al., 1989; Martinez-Argudo et al., 2004). Upon anaerobiosis, NifL is reduced and sequestered to the cytoplasmic membrane, contributing to the release of active NifA (Klopprogge et al., 2002).

NifA is embedded in the global N regulation (*ntr*) signaling cascade at two levels, with both *nifLA* transcription and the stability of the NifLA complex independently coupled to the availability of fixed N (Drummond et al., 1983). During N limitation stress, the two-component system NtrB–NtrC is responsible for expression of the large *ntr* regulon (∼50 genes) associated with N assimilation, scavenging, and growth on alternative N sources (Zimmer et al., 2000). Like NifA, the NtrC response regulator is a σ^54^-type bEBP, which is activated *via* phosphorylation in N limiting conditions. The paralogous PII-type signal transduction proteins GlnB and GlnK are subject to reversible covalent modification by the uridylyltransferase/uridylylremovase (UT/UR) enzyme in direct response to the intracellular glutamine concentration, a key indicator of cellular N status (Merrick, 2015). The PII proteins also report on cellular carbon status *via* allosteric interactions with 2-oxoglutarate (α-KG) and ATP. When intracellular glutamine is low, the trimeric PII-type proteins are uridylylated and stimulate phosphorylation of NtrC by NtrB. Activated NtrC initiates transcription of the *nifLA* operon, in addition to *glnA* [encoding the glutamine synthetase (GS) enzyme essential for ATP-dependent assimilation of ammonium at low concentrations], *amtB* (ammonium transporter channel), and *glnK*. Conversely, the sensing of high glutamine levels in the presence of replete ammonium, the preferred N source for rapid growth, results in de-phosphorylation of NtrC and stringent repression of the Ntr regulon (Schumacher et al., 2013). GlnK expression is induced by N limitation whilst GlnB is constitutively expressed, with the result that the PII proteins are believed to fulfill distinct roles in N regulation (Dixon and Kahn, 2004; Gosztolai et al., 2017). GlnK specifically exerts the second layer of N-mediated control over *nif* gene expression through its interaction with the NifLA complex. Evidence suggests that in *Ko*, GlnK is required to relieve NifA of inhibition by NifL (Jack et al., 1999), independently of its uridylylation state (He et al., 1998). NifA is constitutively active in the absence of NifL (Kim et al., 1989), highlighting the importance of the anti-activator in integrating physiological signals of N limitation (GlnK) and anaerobiosis (redox state) in the control of *nif* expression (Martinez-Argudo et al., 2004).

Re-wiring the metabolism of free-living soil diazotrophs to achieve excretion of surplus fixed N in the plant root environment represents a short-term alternative to the two ambitious synthetic biology strategies being explored for the development of N-fixing crops (Batista and Dixon, 2019): engineering N-fixing symbioses in non-leguminous crops (Rogers and Oldroyd, 2014) and expression of nitrogenase function directly in plant cells (Burén et al., 2018). Mutant diazotroph strains defective in the regulation of N metabolism have proved effective in plant growth promotion in N limited soils (Iniguez et al., 2007; Setten et al., 2013; Barney et al., 2015; Pankievicz et al., 2015; Ambrosio et al., 2017) and are now a key constituent of newly emerging commercial agricultural inoculants (Bloch et al., 2020; Temme et al., 2020).

Essential to these ongoing biotechnological approaches is the understanding and repurposing of the *Ko nif* regulon, which has previously been refactored using standardised synthetic parts for robust heterologous control (Temme et al., 2012; Smanski et al., 2014), reorganised into large cleavable polyproteins for stoichiometric expression in eukaryotic hosts (Yang et al., 2018) and partially expressed in the plant mitochondrial matrix (Allen et al., 2017). However, there are fundamental aspects of the free-living diazotrophy adaptation still to be understood, such as how cells can afford the significant protein investment in nitrogenase under N limitation stress. A long evolved and complex regulatory network exists to maximise cell growth over a wide range of N sources, likely preventing adaptive resource investment in non-optimal conditions and minimizing leakage of fixed N into the environment. This implies that perturbing N metabolism has the potential to yield significant fitness penalties in competitive soil environments. A mechanism by which both fixed N excretion and competitive fitness can be achieved has yet to be revealed (Batista and Dixon, 2019).

To provide a basis to rationally engineer diazotrophy in bacteria, here we present a multi-omics framework, exploiting quantitative proteomics, targeted metabolite estimates, and transcriptome analyses, with which to model N economy in *Ko*. By focusing on the transition from N-replete to diazotrophic growth states, we explore how the N regulatory network enables adaptation to transient fluctuations in extracellular N similar to those experienced in natural environments.

## Materials and Methods

### Bacterial Strains and Plasmids

The bacterial strains, plasmids, and oligonucleotides used in this study are described in Supplementary Table 1. *E. coli* and *Ko* strains were grown in Lysogeny Broth (LB) at 37 or 30°C, respectively, for general propagation, cloning, and transformations. The *Ko* M5a1 strain was obtained from Ruth Schmitz (Christian Albrechts University, Kiel). The Δ*nifLA*, Δ*glnK*, and Δ*glnB* knockout mutants and *nifK*_1__–__1203_ truncation mutant were derived by Lambda Red recombineering (Datsenko and Wanner, 2000). Oligonucleotides were designed in order to amplify a kanamycin resistance cassette (*nptII*) from the pGEM-T-KanFRT plasmid (Zumaquero et al., 2010) with 60 nt overhangs homologous to the flanking regions of the *Ko* M5a1 genomic region targeted for knockout mutagenesis (pGEM-T-KanFRT binding region in upper case in Supplementary Table 1). PCR was performed using Phusion polymerase (Thermo Scientific) with 25 ng template plasmid and an annealing temperature of 55°C. Template DNA was removed by DpnI digestion prior to gel extraction of products. *Ko* M5a1 was transformed with the pKD46 plasmid, expressing the Lambda Red genes required for homologous recombination under the control of an arabinose-inducible promoter. Competent Red-recombinase expressing cells were prepared in super optimal broth (SOB) [0.5% (w/v) yeast extract, 2% (w/v) tryptone, 10 mM NaCl, 2.5 mM KCl, 20 mM MgSO_4_], to which L-arabinose was added to a final concentration of 10 mM once OD_600_ reached 0.1. Cells were grown to an OD_600_ of 0.4–0.6 before being washed three times in sterile cold 10% glycerol solution. Approximately 1 μg of purified PCR product was incubated with 100 μL of competent cells on ice for 30 min prior to electroporation. Transformed cells were recovered at 30°C for 3 h before being plated out on LB agar plates supplemented with kanamycin. Kanamycin-resistant colonies were used to generate seed cultures from which genomic DNA was extracted. Locus-specific homologous recombination was confirmed by diagnostic PCR, combining primers specific to regions flanking the knockout locus with those specific to the *nptII* cassette, and Sanger sequencing.

### Diazotrophic Growth

NH_4_^+^ run-out was used to de-repress *gln*/*nif* gene expression and achieve a reproducible transition into diazotrophic growth. *K. oxytoca* strains were cultured in Nitrogen-Free David and Mingioli (NFDM) medium [69 mM K_2_HPO_4_, 25 mM KH_2_PO_4_, 0.1 mM Na_2_MoO_4_, 90 μM FeSO_4_, 0.8 mM MgSO_4_, 2% (w/v) glucose] supplemented with NH_4_Cl as a nitrogen source. To ensure replete cellular N status, seed cultures were supplemented with 20 mM NH_4_Cl and grown to an OD_600_ of approximately 2–3. Following centrifugation at 4000 × *g* and 4°C, cell pellets were washed once and resuspended in NFDM supplemented with 0.5 mM NH_4_Cl to an OD_600_ of 0.1. Where required, N-replete controls were prepared similarly but supplemented with an additional 10 mM NH_4_Cl. For small-scale growth assays, 200-μL cultures were incubated in triplicate in sterile 96-well culture plates and OD_600_ was measured at 10-min intervals using a CLARIOstar plate reader (BMG LABTECH), in which a microaerobic atmosphere (0.2% O_2_, 1% CO_2_, and 98.8% N_2_) was maintained using an atmospheric control unit and gas cylinders. For batch culture experiments, 100 mL cultures were sealed in 150-mL Erlenmeyer flasks using Suba-Seal septa (Sigma-Aldrich) and chilled on ice to slow cell growth and NH_4_Cl depletion whilst flushed with N_2_ gas for 45 min. Colorimetric O2xyDot sensors (OxySense) fixed inside the flasks were used to verify micro-aerobic conditions. Cultures were warmed to 25°C and shaken at 200 rpm for up to 24 h. To sample cell culture, an equivalent volume of oxygen free N_2_ gas was injected into the headspace *via* gas-tight syringe and the flask inverted in order to extract cells and medium. All experiments were performed with three independent biological replicate cultures for each strain or condition.

### Acetylene Reduction Assay

One milliliter pure, O_2_-free acetylene (BOC) at room temperature and pressure was injected into air-tight culture vessels at the beginning of run-out experiments (*t* = 0 h). The partial pressure of acetylene used was sub-saturating and approximately equivalent to the *K*_*m*_ of the nitrogenase (Harris et al., 2018). Five hundred microliters of culture headspace was sampled *via* gas-tight syringe at 2.5-h intervals, immediately prior to cell culture sampling, and subject to gas chromatography through a HayeSep N column (Agilent) at 90°C in N_2_ carrier gas. Acetylene and ethylene were detected by flame ionisation (FID) at 300°C and ChemStation software (Agilent) was used to integrate signal peak areas. Pure standards of acetylene and ethylene were injected prior to samples to confirm retention times and a Δ*nifLA* culture was used as a negative control. The rate of ethylene production per unit cell density (nmol ethylene OD_600_^–1^ mL^–1^ h^–1^) was estimated using a standard calibration curve (data not shown). Dilution of ethylene as a result of culture sampling was corrected for by measuring the concomitant dilution of acetylene.

### Ammonium Assay

The concentration of NH_4_^+^ ions in 200 μL centrifuged culture supernatant was determined semi-quantitatively using the colorimetric MColorTest kit (Merck). A color change from yellow to blue was measured by absorbance at 690 nm and NH_4_^+^ concentration calculated against a standard calibration curve (78–5000 μM).

### Transcriptome Analysis *via* RNA-Seq

Micro-aerobic *Ko* M5a1 cell cultures were prepared as described above (see section “Diazotrophic Growth”). Briefly, duplicate cultures with an OD_600_ of 0.1 were prepared from an N-replete seed culture and supplemented with 0.5 mM (N run-out) or 10 mM (N-replete) NH_4_Cl, respectively. Once N-fixation was confirmed by acetylene reduction (*t* = 8 h), diazotrophic cell samples were fixed with a 1/10 volume of 5% phenol/95% ethanol (v/v), harvested by centrifugation and stored on dry ice. A single sample of N-replete cells was harvested early during exponential phase (*t* = 1.5 h). Whole-cell RNA extraction, library preparation and quality control, next-generation sequencing, and read alignment was performed commercially by Vertis Biotechnologie AG (Freising, Germany). Cell samples were treated with lysozyme for 15 min in order to extract total RNA, which was then isolated using the mirVana microRNA isolation kit (Ambion). DNase was used to specifically remove contaminant DNA and ribosomal RNA was depleted using the Ribo-Zero rRNA Removal Kit for bacteria (Illumina). The primary RNA transcripts were fragmented using ultrasound (four pulses of 30 s each at 4°C) prior to ligation of 3′ oligonucleotide adapters. First-strand cDNA synthesis *via* Moloney murine leukemia virus (M-MLV) reverse transcriptase was primed using the 3′ adapter. Following purification of first-strand cDNA, a 5′ Illumina TruSeq sequencing adapter (Illumina), inclusive of an 8 nt barcode sequence, was ligated to the 3′ end of the antisense cDNA prior to PCR-amplification of double-stranded cDNA to about 10–20 ng/μL (12–13 thermocycles) using a high-fidelity DNA polymerase. cDNA was purified, pooled in equimolar quantities, and size-fractionated (200–500 bp) using the Agencourt AMPure XP kit (Beckman Coulter Genomics) prior to analysis by capillary electrophoresis. The cDNA pool was sequenced strand-specifically on an Illumina NextSeq 500 system, utilising a read length of 75 nt and a depth of 100 million reads. The output reads were trimmed to remove adapter sequences and filtered to remove duplicates, prior to alignment to the *Ko* M5a1 genome (GenBank WGS accession JAFHKG010000000; BioSample SAMN17288411). Data was provided by Vertis Biotechnologie in the form of raw reads (fastq), binary alignment map (bam) files, and normalised gene counts (reads per kilobase per million, RPKM) (Mortazavi et al., 2008). Raw and processed datasets are accessible *via* the NCBI Gene Expression Omnibus (GEO Series accession GSE164668). Across all samples, between 22.0 and 25.1 million reads (81.4–87.7%) were mapped to the genome, including 56.7–68.5% mapped to annotated genes. Alignments were visualised manually using Integrative Genomics Viewer (IGV) (Robinson et al., 2011). Differential gene expression analysis was performed using the R/Bioconductor statistical package DEseq2 (Love et al., 2014), utilising settings of regularised logarithm (rlog) normalisation of read counts, Wald hypothesis testing, and *p*-value adjustment using a False Discovery Rate threshold of 0.05 (Benjamini and Hochberg, 1995). Log_2_ fold changes between conditions were calculated for differentially expressed genes (DEGs) with an adjusted *p*-value of <0.05. DEG lists were mapped to the KEGG Orthology database using the KEGG Automatic Annotation Server (Moriya et al., 2007), using translated coding sequences as queries for the bi-directional best-hit (BBH) method of ortholog assignment.

### Total Protein and Metabolite Extraction

A methanol-chloroform extraction procedure (Sapcariu et al., 2014) was used for simultaneous, reliable, and medium-throughput extraction of total cell protein and polar metabolites. Fifteen milliliters cell culture (at variable OD_600_) was harvested by centrifugation at 4000 × *g* and 4°C and the pellet rapidly frozen in liquid nitrogen. Cell sample was thawed and resuspended in 800 μL ice-cold 50% (v/v) methanol, followed by the addition of 400 μL ice-cold chloroform. The mixture was agitated at 1000 rpm for 10 min in a cooled TissueLyser II cell disruptor (Qiagen) prior to phase separation by centrifugation at 15,000 × *g*. Seven-fifty microliters of the upper polar phase was extracted and evaporated in a rotary vacuum evaporator until dry, prior to storage at −80°C. The protein interphase was washed in 50% methanol and vortex solubilised in 500 μL urea-phosphate buffer (7 M urea, 69 mM K_2_HPO_4_, 25 mM KH_2_PO_4_). Total protein concentration was determined using the colorimetric DC assay (Biorad) according to manufacturer instructions.

### Liquid Chromatography–Mass Spectrometry

Analyte peptides were prepared by incubating 30 μL total cell protein sample and a mix of protein standards (see below) with 2 μg sequencing grade modified trypsin (Promega) for 5 h at 37°C followed by 20 h at 25°C in a final volume of 300 μL digestion buffer [100 mM Tris–HCl (pH 8), 50 mM NH_4_HCO_3_, 1 mM TCEP]. Ten microliters control samples taken before and after incubation with trypsin were run on SDS-PAGE to verify complete digestion prior to inactivation of trypsin with 2% formic acid. Dried polar metabolites were resuspended in 100 μL 50% methanol prior to a 10 μL aliquot being derivatised using the 6-aminoquinolyl-*N*-hydroxysuccinimidyl carbamate AccQTag Ultra reagent (Waters), as described previously (Gray et al., 2017). Both peptide and amino acid samples were separated by reverse-phase chromatography on a Prominence HPLC system (Shimadzu) and analyzed using a linear ion trap triple-quadrupole 6500 Qtrap mass spectrometer (AB Sciex). Forty microliters peptide sample injections were separated isothermally at 50°C through a Luna C_18_ silica column (Phenomenex, 100 Å pore size, 3 μm particle size, 100 mm × 4.6 mm) with a multi-step solvent gradient: 100% solvent A (94.9% H_2_O, 5% acetonitrile, 0.1% formic acid), equilibration; 0–25% solvent B (94.9% acetonitrile, 5% H_2_O, 0.1% formic acid), 30 min; 25–50% B, 5 min; 50–100% B; 100% B, 5 min. Five microliters derivatised metabolite sample injections were separated isothermally at 45°C and 300 μL per minute through an Acquity T3 C_18_ high-strength silica UPLC column (Waters, 100 Å pore size, 1.8 μm particle size, 100 mm × 2.1 mm) with gradient: 4% B, 0.1 min; 28% B, 10 min; 80% B, 1.5 min; 4% B, 1.5 min. The MS was operated in positive ion mode with the following general parameters: curtain gas, 40 psi; ion source nebulising gas, 40 psi; ion source turbo gas, 60 psi; curtain gas, 40 psi; ion spray voltage, 5500 V; source gas temperature, 500°C; entrance potential, 10 V. The collision cell exit potential was set at a constant 15 V and the retention time detection window at 180–360 s. For MS/MS of amino acids, the following parameters were set as constants: collision energy, 30 V; declustering potential 30 V, collision cell exit potential, 10 V; retention time detection window, 90 s. Transitions lists detailing further analytical parameters specific to peptide and amino acid targets (e.g., Q1/Q3 mass, retention time) are provided in Supplementary Data 1, 3, respectively. Signal and standard peak areas were manually validated and integrated using Skyline software (MacLean et al., 2010) prior to calculation of analyte concentrations and statistical analysis in R. Peptide quantification was achieved by summing the integrated peak areas of two or more validated transitions. Where possible, the abundances of at least two correlative peptides for each protein were averaged to assign protein copy number. Protein copy number per cell values assume an OD-specific cell density of 10^9^ CFU mL^–1^ OD^–1^. Post-translational modification (PTM) values are expressed as the ratio of modified/total (unmodified + modified) states. Amino acid concentrations were calculated relative to external standard curves of a standard mix of 20 amino acids (Cell Free Amino Acid Mixture-^15^N, Sigma-Aldrich) diluted to 0.7–10.0 μM and assuming a similar cell volume of *Ko* M5a1 to that characteristic of *E. coli* (10^–15^ L; Milo, 2013) and an OD-specific cell density of 10^9^ CFU mL^–1^ OD^–1^.

### Design, Purification, and Validation of QconCAT Standard Proteins

Skyline software (MacLean et al., 2010) was used for peptide screening, peak selection, method development, analysis, and data processing purposes. Candidate analytical peptides were selected according to the following criteria: unique to *Ko* M5a1 proteome (extracted from GenBank WGS accession JAFHKG010000000), 5–25 residues in length, 50–1800 *m/z* ratio, and doubly charged with more than three fragment y-ions. Peptides containing His, Met, or Cys residues, proline-adjacent tryptic sites or KK/RR adjacent cut sites were excluded where possible. Unscheduled selected reaction monitoring (SRM) methods were assigned a dwell time of 3 ms per transition and peptide-specific collision energy and declustering potential parameters predicted by Skyline. A total 12–17 candidate peptides verified in test diazotrophic samples (see section “Results”) were included in each QconCAT design, flanked by the three amino acid residues upstream and downstream of the native trypsin cleavage site in order to maintain digestion efficiencies equivalent to the native target proteins. Furthermore, unique quantitative bovine serum albumin (BSA) peptides were incorporated at both termini to act as internal controls against potential degradation by exoproteolysis. Each QconCAT sequence was codon optimised for expression in *E. coli*, synthesised, and inserted into the pET100/D-TOPO expression vector (GeneArt, Life Technologies). For *in vivo* double-labelling at arginine and lysine residues, the His-tagged QconCATs were expressed in a modified Δ*argA* Δ*lysA* BL21 strain (Matic et al., 2011) cultured in Gutnick minimal salts medium (33.79 mM KH_2_PO_4_, 77.51 mM K_2_HPO_4_, 5.74 mM K_2_SO_4_, 0.41 mM MgSO_4_) supplemented with 0.4% glucose, 10 mM NH_4_Cl, 1 mM heavy-labelled L-[^13^C_6_,^15^N_2_]arginine and L-[^13^C_6_,^15^N_2_]lysine (Sigma-Aldrich), and 18 unlabelled amino acids at 1 mM concentrations each. Following sonication, cell protein was extracted from inclusion bodies using 7 M urea. Bench-top column purification of His-tagged protein was performed using Ni-NTA agarose resin with a binding buffer consisting of 95 mM Na_2_HPO_4_, 5.2 mM NaH_2_PO_4_, 50 mM NaCl, and 3 M urea. QconCAT purity was determined by SDS-PAGE. Protein samples were mixed 1:1 with 2 × Laemmli buffer, boiled for 5 min and separated through a 15% polyacrylamide mini-gel over 60 min at 200 mA. Gels were stained with SYPRO Ruby^®^ fluorescent stain and % purity estimated *via* lane-specific densitometry. QconCAT concentration, corrected for purity, was determined *via* DC assay. Following validation of standard response linearity (see section “Results”), QconCATs were added to sample digests at the following concentrations (μg/mL): QNif1, 0.7; QNif2, 0.124; QNif3, 0.081; QNtr, 0.97.

## Results

### NH_4_^+^ Run-Out and Induction of Diazotrophic Growth

**FIGURE 1:**
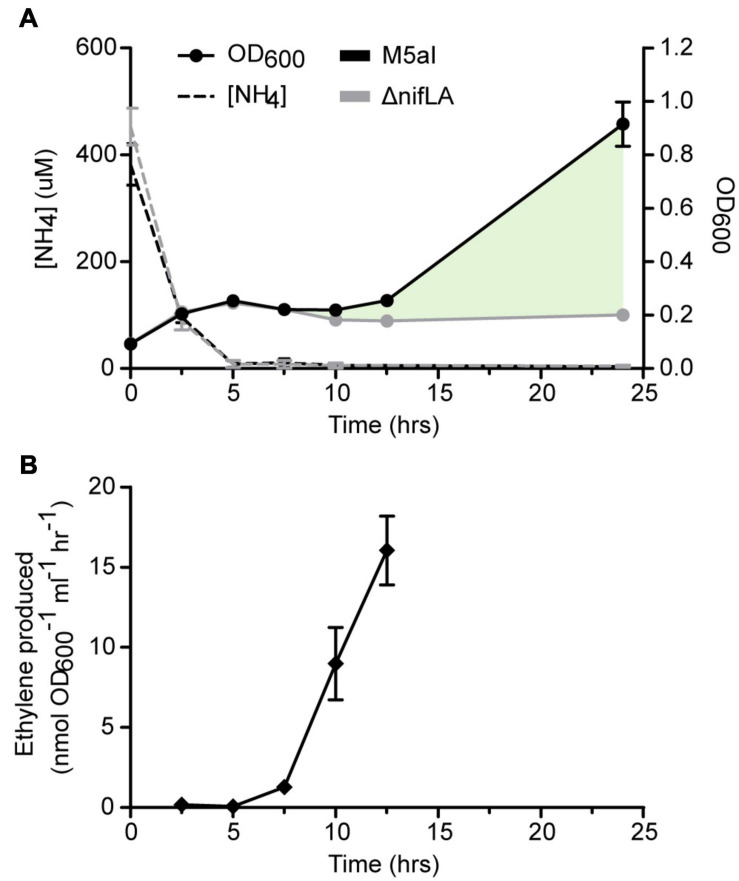
Physiological parameters of NH_4_^+^ run-out and diazotrophy. *Ko* M5a1 wild-type (black) and Δ*nifLA* (gray) strains were cultured for 24 h in NFDM medium supplemented with 0.5 mM NH_4_Cl under micro-aerobic conditions. **(A)** Cell density (OD_600_, filled circles) and supernatant NH_4_^+^ concentration (dashed lines) were measured at 2.5 h intervals and again after 24 h. Cell growth attributable to NifLA expression and diazotrophy is represented by shaded fill (pale green). **(B)** Culture headspace was sampled at 2.5-h intervals (between 2.5 and 12.5 h) for detection of nitrogenase activity *via* the acetylene reduction assay. Acetylene was injected into the head space at *t* = 0 h. Activity values correspond to ethylene evolved per hour and per unit cell density at 25°C. Error bars represent SEM of three biological replicates. Acetylene reduction was not assayed at 24 h.

The bacterial nitrogen stress response was induced experimentally upon metabolic consumption of a limited concentration of ammonium in the growth medium (here termed run-out) (Schumacher et al., 2013; Gosztolai et al., 2017). We supplemented *Ko* M5a1 with an initial ammonium salt concentration (0.5 mM NH_4_Cl) that enabled both nitrogen starvation and, subsequently, diazotrophic growth to be observed over the course of a day culture (approximately 12 h). Cells were pre-cultured overnight in excess NH_4_Cl (20 mM) to ensure a replete cellular N status prior to run-out. The limiting NH_4_^+^ concentration in the culture supernatant was largely depleted (<3% of starting concentration) after 5 h, coinciding with a plateauing of cell growth (Figure 1A). Nitrogenase activity was first detected after 7.5 h *via* the acetylene reduction assay (Figure 1B). An increase in OD_600_ suggests that cell growth slowly resumed between 10 and 12.5 h, during which period nitrogenase activity also increased. An endpoint OD_600_ measurement was taken after 24 h, at which point the cell density of wild-type *Ko* M5a1 had increased by more than threefold with respect to the onset of nitrogen starvation. However, this time point was not considered for further analyses given that the cell state may be strongly affected by secondary variables such as carbon limitation and changing pH of the growth medium. Growth did not resume following nitrogen starvation in a Δ*nifLA* mutant control, confirming that wild-type growth under these conditions is diazotrophic and dependent on nitrogenase expression. The supernatant NH_4_^+^ concentration remained depleted during diazotrophic growth, suggesting that NH_4_^+^ derived from fixation of N_2_ by the nitrogenase is rapidly assimilated and its net excretion (accounting for AmtB-mediated re-uptake) is negligible in the growth conditions used.

### The *nif* and *ntr* Regulons Are Highly Upregulated Early in Diazotrophy

**FIGURE 2:**
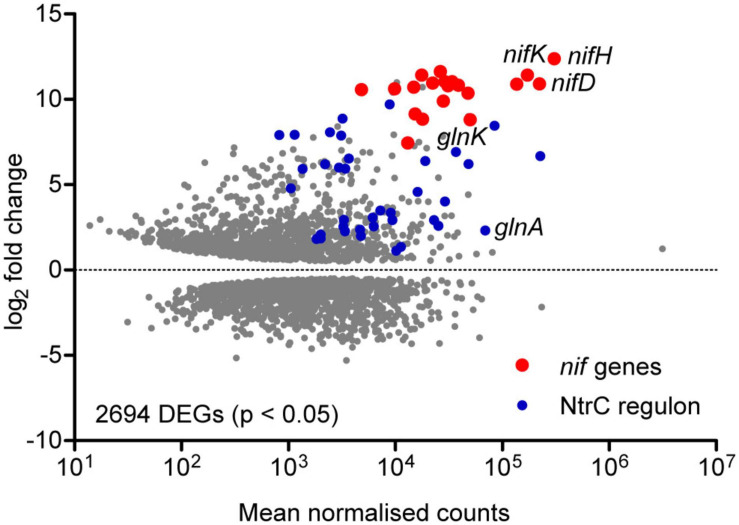
Exploratory analysis of the diazotrophic transcriptome. MA plot of the log_2_ fold change and mean RNA-seq read count for all *Ko* M5a1 genes. Gray points represent differentially expressed genes (DEGs) in N-fixing conditions (0.5 mM NH_4_Cl run-out) with respect to N-replete (10 mM NH_4_Cl) conditions, with adjusted *p*-values of <0.05 as determined by DESeq2. Read counts were averaged (mean) across all N-fixing and N-replete samples and normalised using the regularised logarithm (rlog) transformation. DEGs belonging to the known *nif* and NtrC-dependent regulons are highlighted in red and blue, respectively.

In order to assess the global gene expression profile associated with diazotrophic growth, the *Ko* M5a1 transcriptome in N-fixing (*t* = 8 h; OD_600_ = 0.21, 0.19) and N-replete (exponential growth in 10 mM NH_4_Cl; OD_600_ = 0.24) conditions was compared by RNA-seq. As only one N-replete replicate was sequenced, requiring variance for this condition to be estimated and resulting in reduced statistical power (Anders and Huber, 2010), the analysis presented here is exploratory in nature and focused on genes exhibiting large fold changes. More than 50% of the transcriptome (2694/5279 genes) was highlighted as differentially expressed (adjusted *p*-value < 0.05) under diazotrophic conditions (Figure 2 and Supplementary Data 2). Given that glucose concentration and micro-aerobiosis were constant across both conditions, adaptation to nitrogen stress and its indirect global effects on metabolism and growth was most likely responsible for this concerted reprogramming of transcription. The *nif* genes were both highly upregulated and highly expressed in the diazotrophic condition. Normalizing for transcript length (RPKM), *nifH* was the most highly expressed gene and all 20 *nif* genes were among the 200 most highly expressed genes by rank. Nine hundred and twenty-five genes were upregulated and 1117 downregulated by more than twofold. Of these, 1515 correspond to annotated proteins in the KEGG functional orthology (KO) database, including metabolic enzymes, gene expression machinery, and transporters (Supplementary Figure 1). In addition to the *nif* genes, numerous NtrC-dependent genes associated with the bacterial nitrogen stress response were upregulated, including *glnA* (GS), *glnK* (PII-type signaling protein), the *rut* (pyrimidine scavenging), and *ast* (arginine catabolism) operons and several operons encoding amino acid transporters. Operons encoding enzymes and transporters required for metabolism of nitrate/nitrite (KO: NrtABC-NirB-NasA) and urea (KO: UreABC-UrtABCDE) and purine scavenging (KO: HpxAB-HpxO-HpxWXYZ) were also highly upregulated. Whilst these operons are not associated with the canonical NtrC regulon described in *E. coli* (Zimmer et al., 2000), analysis of the promoter sequences upstream of their apparent transcriptional start sites suggests that NtrC and σ^54^ may play a role in their expression in *Ko* M5a1 (data not shown). This contrasts with the operon encoding a branched-chain amino acid transport system (KO: LivKHMGF) which is highly upregulated but exhibits no obvious promoter NtrC binding sites. Whilst *glnA* is highly expressed, the downregulation of *gdh* by more than 10-fold is consistent with the periphery role of glutamate dehydrogenase (with a far lower affinity for ammonium than GS) in nitrogen assimilation during N limitation stress conditions. The array of other downregulated genes observed may reflect the wider impacts of nitrogen starvation on metabolism, growth, and phenotype. Downregulation of genes associated with DNA replication, RNA transcription, and purine (*pur*) and pyrimidine (*pyr*) nucleotide biosynthesis implies a decreased resource allocation into cell division and gene expression. Similarly, downregulation of 48 ribosomal proteins, 35 ribosome biogenesis genes and 55 tRNA biosynthesis genes may reflect a partial hibernation of the protein translation machinery. This hypothesis is supported by the upregulation of *raiA* and *sra*, encoding stationary phase-associated ribosome inhibitors, by more than 15- and 4-fold, respectively. General reprogramming of carbohydrate metabolism, amino acid metabolism, and membrane transport (ABC) is reflected by similar numbers of upregulated and downregulated genes in these functional groups. However, it is noted that many genes may be differentially expressed due to indirect effects of N starvation on metabolism and growth rather than active regulation in response to starvation.

In order to assess the extent of post-transcriptional regulation in *nif* gene expression, the sequencing read mapping profile of the *nif* cluster was examined at nucleotide resolution for signatures of antisense transcription or variation in mRNA stability (Supplementary Figure 2). No evidence of antisense transcription was observed within any of the eight known *nif* transcriptional units. Each transcriptional unit was defined by a 5′ peak in read density commencing 12 bp downstream of a putative sigma-54 recognition sequence (consensus TGGC-N_7_-TTGC). The notable spike in read density observed at the end of the *nifHDKTY* operon raises the possibility of an internal secondary promoter for *nifY*.

### Development and Validation of QconCAT Standards for the Nif and Ntr Regulons

**FIGURE 3:**
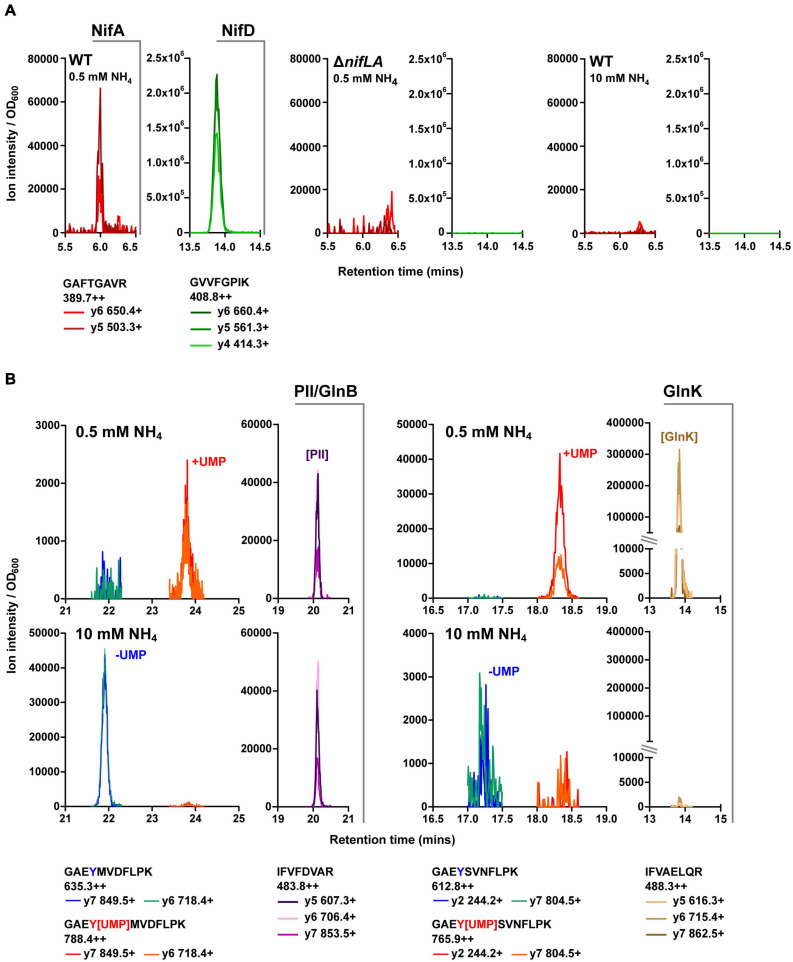
Validation of key Nif and Ntr peptides. Retention time specific LC–MS peaks corresponding to candidate peptides (see Supplementary Data 1) in representative biological test samples: diazotrophic (wild-type, 0.5 mM NH_4_Cl), non-diazotrophic/N limiting (Δ*nifLA*, 0.5 mM NH_4_Cl) and N-replete (wild-type, 10 mM NH_4_Cl). Multiple daughter y-ion fragments (+) are shown for each parent ion (++), along with the corresponding *m/z* values. Peak ion intensity is normalised to sample OD_600_ for relative quantification between samples. **(A)** NifD and NifA. **(B)** GlnB and GlnK. Non-modifiable peptides are indicative of relative PII-protein abundance between samples. Peaks at discrete retention times represent uridylylated (+UMP; red/orange) and non-uridylylated (–UMP; blue/green) forms of modifiable peptides. Note variable *y*-axis scales.

Selected reaction monitoring-MS was utilised for targeted quantification of the *Ko* M5a1 proteome compartment associated with N metabolism and its regulation during diazotrophic growth. Furthermore, the QconCAT approach, highly suited to SRM workflows, was adopted for development of multiplexed standards for absolute quantification (Supplementary Figure 3). Protein sequences of interest were subjected to *in silico* trypsin digest and candidate analytical peptides were selected for empirical validation. At least three parent–daughter ion transitions for each candidate peptide were assimilated into a series of unscheduled SRM methods for screening against biological samples. Total cell protein was isolated from three sample cell cultures representing different expression states of the N proteome: (i) *Ko* M5a1, 5 h after NH_4_^+^ run-out (N stress and Nif expression); (ii) Δ*nifLA*, 5 h after NH_4_^+^ run-out (N stress but no Nif expression); (iii) *Ko* M5a1, supplemented with 10 mM NH_4_Cl (N-replete). Trypsin digested samples were analyzed by SRM-MS and peptides exhibiting multiple co-eluting transition peaks in the diazotrophic sample (i) but a low background in the corresponding negative control sample (ii and iii for Nif peptides; iii for Ntr peptides] were selected for further QconCAT standard development. The *nifLA*-dependent and N stress-dependent detection of SRM transitions specific to exemplar NifA (GAFTGAVR) and NifD (GVVFGPIK) peptides is outlined in Figure 3A. An enhanced product ion (EPI) scan was performed to confirm the identity of peptides with low peak intensity (data not shown). Empirical screening identified signature proteotypic peptides for 19 of the 20 Nif proteins. All four candidate peptides for NifV were undetectable in all samples.

Peptides we have previously shown to be quantotypic for the Ntr signaling proteins NtrC, GlnB, GlnK, and the GS enzyme in *E. coli* (Schumacher et al., 2013; Gosztolai et al., 2017) exhibit high sequence identity with their *Ko* M5a1 homologues. Pairs of post-translationally modifiable (containing the uridylylated Y51 residue) and non-modifiable peptides of the PII-type proteins GlnB and GlnK were analyzed for their utility in reporting on both N stress-dependent protein expression level and uridylylation state. The modifiable peptides were detected predominantly in their uridylylated forms in N-limiting conditions and their non-uridylylated forms in NH_4_Cl-replete conditions (Figure 3B). The covalent addition of the uridine monophosphate (UMP) moiety resulted in an increase in peptide retention time of approximately 2 min for both GlnB and GlnK. Whilst the non-modifiable peptide of GlnB was observed at similar intensities in both cellular N states, the equivalent GlnK peptide was only detected in the N-limiting condition. This is consistent with the expression patterns of the PII-type proteins in *E. coli*, whereby *glnK* transcription is governed by NtrC activity while *glnB* expression is constitutive.

We designed four QconCAT sequences (QNif1, QNif2, QNif3, QNtr; see Supplementary Data 1) encompassing a total of 55 validated signature peptides, with at least two per target protein. Purified and heavy-labelled (^13^C^15^N-arginine and lysine) QconCATs were purified (Supplementary Figure 4) and mixed with cell protein samples (i, ii, iii), prior to trypsin digest and SRM-MS for sample peptide validation and SRM method scheduling. The enzymatic cleavage of all signature peptides from the QconCAT standards was confirmed (Supplementary Figure 5) and the absence of unlabelled peptide peaks in standard-only control samples confirmed completeness of *in vivo* labelling. The linearity and limit of detection for each peptide was assayed by titrating a fixed concentration of QconCATs with a dilution series (10^0^–10^–5^) of cell protein extract (Supplementary Figure 6). SRM transitions exhibiting non-linearity or a poor limit of detection were excluded during method optimisation. The absolute quantification of Nif protein copy numbers in diazotrophic cells, inferred from the unlabelled/labelled (sample/QconCAT) ratio of SRM signal peak area and QconCAT standard concentration, was largely consistent across independent signature peptides (Supplementary Figure 7). Besides NifQ and NifZ, which exhibited no linear peptides, each Nif protein exhibited at least one peptide with a limit of detection below 1 nM in the digested sample.

### The Protein Stoichiometry of the Nif Regulon

**FIGURE 4:**
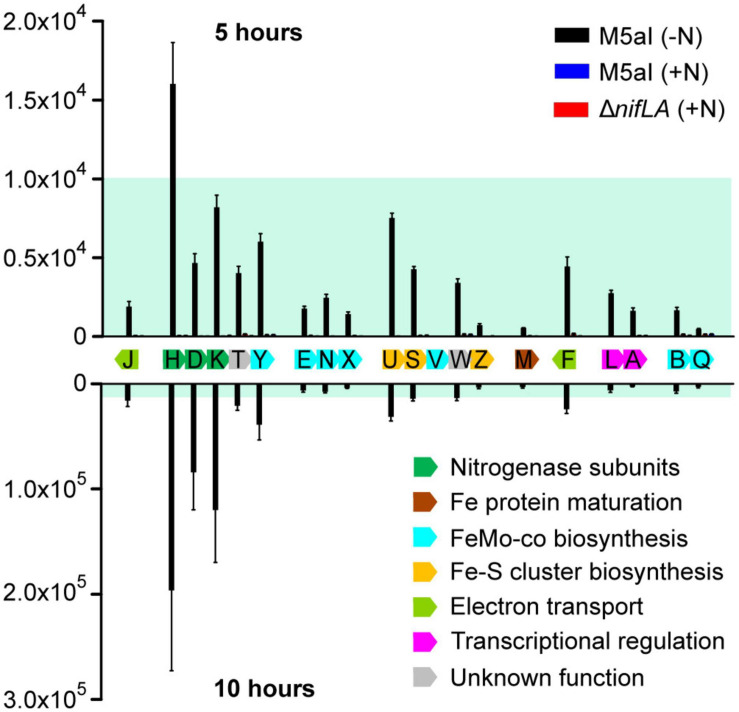
Stoichiometry of Nif proteins during the onset of diazotrophy. Absolute copy number of 19 detectable Nif proteins at the onset of nitrogen starvation (5 h following supplementation with 0.5 mM NH4; above the *x*-axis) and at the onset of slow diazotrophic growth (10 h; below the *x*-axis) in *Ko* M5a1 wild-type cells (black bars). N-replete conditions (wild-type plus 10 mM NH_4_Cl; blue bars) and Nif– (Δ*nifLA*; red bars) cells were used as negative controls, which are barely visible in the figure. Protein samples were supplemented with heavy-labelled QconCAT standards (QNif1, QNif2, QNif3) prior to tryptic digestion. Multiple peptide transitions per Nif protein (see Supplementary Data 1) were integrated for calculation of absolute protein copy number per cell. Estimates inferred from known QconCAT concentrations and the mean sample/QconCAT LC–MS peak area ratio, assuming an OD_600_ of 1.0 = 10^9^ CFU mL^–1^ and a cell volume of 10^–15^ L. Error bars represent SEM of three biological replicates. NifV was undetectable in all samples. Nif proteins are displayed according to genomic organization and colored by function. Blue shading representative of ≤10,000 copies and highlights variable *y*-axis scale.

Figure 4 shows the cellular copy number of the 19 quantifiable Nif proteins at two time points during the adaptation to diazotrophy, corresponding to (a) onset of nitrogen starvation (5 h following supplementation with 0.5 mM NH_4_) and (b) onset of slow diazotrophic growth (10 h). Negligible Nif protein concentrations, below the limits of detection, were observed in the two control conditions (Δ*nifLA*; wild-type supplemented with 10 mM NH_4_Cl), highlighting the strong dependence of *nif* promoter activation on both NifA and nitrogen limitation. Although absolute quantification cannot reveal the fraction of any given protein that is either functional or in a specific complex, the observed NifH/NifDK ratio of approximately 2 closely reflects the known *in vivo* catalytic stoichiometry of the nitrogenase enzyme (Peters et al., 1995). Likewise, the abundances of the NifL and NifA regulators were broadly similar, with a modest excess of NifL suggesting that all NifA activator proteins are able to be sequestered by NifL acting as an inhibitor. Whilst the stoichiometry of the nitrogenase subunits remained constant over time, the ratio of the nitrogenase complex to the accessory Nif proteins (involved in co-factor biosynthesis, assembly, and regulation) increased between 5 and 10 h (Supplementary Figure 8). This pattern of accumulation may reflect both the order in which the Nif protein functions are required for diazotrophy to be established and the relative rates of different steps/reactions required. For instance, the accessory protein NifU was the third most abundant Nif protein at 5 h, consistent with its role in the synthesis of Fe–S clusters for both nitrogenase structural (NifH) and co-factor biosynthesis (NifB) proteins (Jimenez-Vicente et al., 2018), but does not accumulate further at a similar rate to NifHDK.

### Time-Resolved Expression of Nitrogenase and GS in Response to N Stress Signaling

**FIGURE 5:**
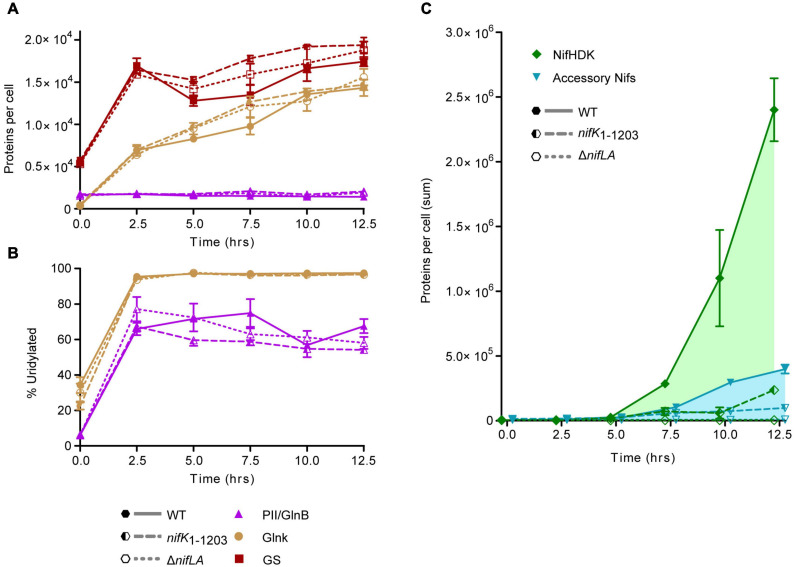
Time-course of Nif and Ntr protein expression and post-translational N stress signaling. *Ko* M5a1 wild-type (full lines, full shapes) *nifK*_1__–__1203_ (dashed lines, half shapes) and Δ*nifLA* (dotted lines, empty shapes) strains were cultured for 12.5 h in NFDM medium supplemented with 0.5 mM NH_4_Cl under micro-aerobic conditions. Cell protein was extracted at 2.5 h intervals, supplemented with a mix of four QconCAT standards and trypsin digested. Absolute copy number was inferred from the sample/QconCAT LC–MS peak area ratio for quantotypic non-modifiable peptides and QconCAT concentration, assuming an OD600 of 1.0 = 10^9^ CFU mL^–1^ and a cell volume of 10^–15^ L. Error bars represent SEM of three biological replicates. **(A)** Copy number of Ntr proteins: GlnB (purple), GlnK (brown), and glutamine synthetase (GS) (orange). **(B)** Post-translational modification of PII-type proteins GlnB and GlnK. Percentage uridylylation was inferred from the modified/total LC–MS peak area ratio for modifiable peptides. **(C)** Summed absolute copy numbers of nitrogenase subunits NifHDK (green) and accessory proteins associated with co-factor biosynthesis and assembly (blue). Overlapping data points offset for clarity.

Absolute Nif protein expression was correlated with cellular nitrogen status throughout a complete time course encompassing NH_4_^+^ run-out and subsequent diazotrophic growth, as in Figure 1. In order to relate the benefit of N fixation activity to the cost of Nif expression, protein samples were extracted in parallel from two non-diazotrophic strains representative, at the level of protein expression, of Nif+ (*nifK*_1__–__1203_) and Nif− (Δ*nifLA*) states alongside wild-type (diazotrophic and Nif+). The *nifK*_1__–__1203_ mutant was constructed such that a C-terminal domain of NifK necessary for catalytic activity of the nitrogenase is lacking but *nif* expression otherwise unimpaired. Complete protein copy number data are provided in Supplementary Data 1. Protein signatures of N limitation stress were evident prior to complete NH_4_^+^ starvation in all three strains, with increases in both NtrC-dependent protein expression (GlnK and GS) (Figure 5A) and the percentage of GlnB and GlnK protein uridylylation (Figure 5B) between 0 and 2.5 h. This response coincided with a supernatant NH_4_^+^ concentration of approximately 100 μM at 2.5 h, prior to depletion to a level representative of complete starvation (<10 μM) by 5 h (see Figure 1). GlnK levels increased more than 40-fold over the course of the diazotrophy adaptation whilst GlnB protein levels remained constant, consistent with *glnB* being constitutive and regulated independently of NtrC activity. At the onset of N starvation (*t* = 5 h), the wild-type copy numbers of GlnK and GlnB were 8200 and 350 per cell, respectively. GS increased approximately threefold during NH_4_^+^ run-out, peaking at approximately 17,000 copies per cell (*t* = 2.5 h).

Both PII-type signaling proteins were highly uridylylated during NH_4_^+^ runout and remained in this PTM state for the duration of the diazotrophy adaptation. Furthermore, there was no notable difference in PTM status between the wild-type and the non-fixing Δ*nifLA* and *nifK*_1__–__1203_ strains. These findings imply that diazotrophy does little to relieve the cells perceived N limitation stress, which remains a persistent characteristic of the diazotrophic growth state. However, there was a marked difference between the absolute levels of GlnK and GlnB uridylylation, both in N starved and N-replete conditions. Whilst >95% of GlnK was uridylylated following N starvation, the maximal level of GlnB uridylylation observed was around 75%, peaking around the onset of detectable nitrogenase activity in the wild-type and prior to complete NH_4_^+^ run-out in Δ*nifLA* and *nifK*_1__–__1203_. In N-replete conditions (*t* = 0 h) where GlnB outnumbers GlnK approximately fourfold, their uridylylation levels were approximately 5 and 35%, respectively. It is also notable that the level of GlnB uridylylation decreased following the onset of diazotrophy in the wild-type (*t* = 7.5–10 h) and steadily throughout prolonged N starvation in the non-diazotrophic strains. Taken together, these observations suggest that uridylylated GlnK is the by far the most abundant PII isoform during diazotrophy, whilst GlnB is the more sensitive of the two proteins to finer shifts in N status.

The detection of Nif proteins at levels exceeding limits of detection coincided with the onset of N starvation (*t* = 5 h). The time-resolved expression of the individual Nif proteins is shown in Supplementary Figure 9. As observed in previous experiments (see Figure 4), the nitrogenase subunits NifHDK accumulated more rapidly than the accessory Nif proteins (Figure 5C). Between 10 and 12.5 h, the concentration of nitrogenase subunits more than doubled, whilst the sum of the other Nif proteins increased by only 35%. Assuming that the concentration of fully assembled nitrogenase complex is limited by the least abundant of the individual NifHDK proteins, it can be inferred that the nitrogenase increased more than 50-fold (800–50,000 enzymes per cell) between the onset of N starvation (5 h) and the resumption of slow diazotrophic growth (12.5 h). Nitrogenase far exceeded the concentration of the GS complex, which does not exceed 1500 molecules per cell (taking into account its 12 subunit assembly) throughout N starvation. The apparent disparity in concentration may compensate somewhat for the significantly different turnover rates for nitrogenase (∼5 s^–1^) and GS (>500 s^–1^; van Heeswijk et al., 2013). It is notable that the NtrC-dependent gene products (GlnK, GS) accumulated several hours prior to the NifA-dependent *nif* gene products, suggesting that the two regulons are induced at different thresholds of N stress.

The total abundance of NifHDK and accessory Nif proteins in the non-diazotrophic *nifK*_1__–__1203_ mutant fell to 10 and 25% of wild-type levels, respectively, by 12.5 h into the time course (Figure 5C). This disparity between the strains was not evident at the 5 h time point (Supplementary Figure 9F), implying that it was not the result of differences in the kinetics of *nif* gene de-repression in response to N starvation. Rather, the exaggeration of the *nifK*_1__–__1203_ phenotype throughout the diazotrophy transition suggests that N fixation function is required to sustain further Nif protein expression and, by implication, that stored cellular biomass derived from nutrients in the growth medium alone is insufficient to support the expression levels observed in the wild-type. As expected, no evidence of Nif protein expression was observed in the Δ*nifLA* mutant throughout the time course. Despite the difference in Nif protein burden, however, no significant difference in cell density between Δ*nifLA* and *nifK*_1__–__1203_ was evident (Supplementary Figure 9G). This may reflect the fact that neither strain can undergo diazotrophic growth following N starvation and therefore any fitness penalty due to futile Nif expression in *nifK*_1__–__1203_ is not manifest in this respect.

### Adaptation of the Amino Acid Pool to N Starvation and Diazotrophy

To explore whether the burden of Nif protein expression and N stress more broadly are manifest at the level of substrate amino acids, we quantified the intracellular pools, extracted simultaneously with total protein, in the wild-type, Δ*nifLA*, and *nifK*_1__–__1203_ strains *via* LC–MS. The limit of quantitation of each amino acid was determined *via* a dilution series of amino acid standards of known concentration (0.1–100 μM). MS signal response was linear for all amino acids, with robust quantitation at 0.1 μM for all except asparagine, aspartate, and glutamine, for which signal response was linear at 2 μM and above (Supplementary Figure 10A). The calculated amino acid pool concentrations for NH_4_^+^ replete cells sampled immediately following transfer to 0.5 mM NH_4_^+^ medium were consistent with those observed in *E. coli* cells grown in similar N-replete conditions (Supplementary Figure 10B; Bennett et al., 2009), indicative of effective metabolite extraction and quantification.

**FIGURE 6:**
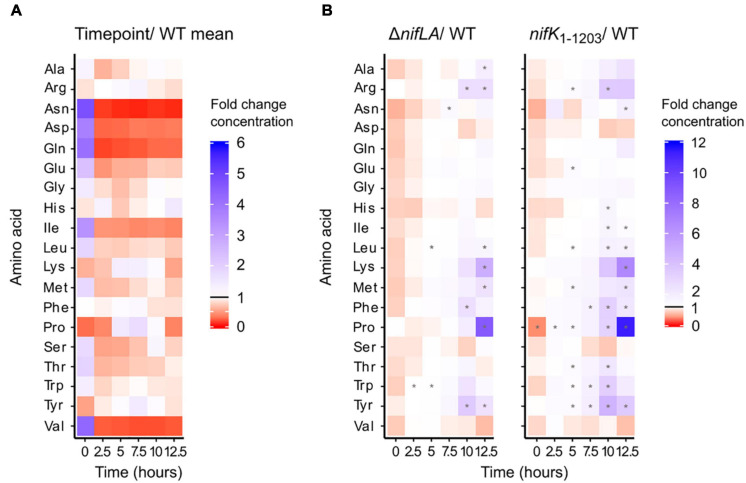
Dynamics of the intracellular amino acid pools during the diazotrophy transition. *Ko* M5a1 wild-type, *nifK*_1__–__1203_, and Δ*nifLA* strains were cultured for 12.5 h in NFDM medium supplemented with 0.5 mM NH_4_Cl. Polar metabolites were extracted at 2.5 h intervals, derivatised with the AccQTag reagent (6-aminoquinolyl-*N*-hydroxysuccinimidyl carbamate) and analyzed *via* LC–MS. Absolute amino acid concentrations were calculated by referencing external standard curves, assuming a characteristic cell volume of 10^–15^ L. **(A)** Time course of wild-type amino acid pools. Heat map density representative of fold-change between time-specific concentrations and the base mean amino acid concentration across all time points. Asterisks denote statistically significant differences from base mean (one sample unpaired Student’s *t*-test, *N* = 3, *p* < 0.05). **(B)** Time course of Δ*nifLA* and *nifK*_1__–__1203_ amino acid pools. Heat map density representative of fold-change between Δ*nifLA* and *nifK*_1__–__1203_ amino acid concentrations and wild-type. Asterisks denote statistically significant differences between mutant and wild-type means (two sample unpaired Student’s *t*-test, *N* = 3, adjusted *p*-value (False Discovery Rate; Benjamini and Hochberg, 1995; *p* < 0.05). Note different heat map density scales between panels **(A,B)**. Absolute amino acid concentrations are detailed in Supplementary Data 3.

Absolute amino acid concentrations varied by several orders of magnitude during the wild-type transition to diazotrophy, from a high of 70 mM glutamate in N-replete conditions to a low of 5 μM histidine following NH_4_^+^ run-out. Total amino acid concentrations are shown in Supplementary Figure 11 and listed individually in Supplementary Data 3. To provide a comparative overview of how amino acid pool sizes respond to N starvation and the onset of diazotrophy, absolute concentrations were normalised against the base mean for each amino acid (Figure 6A). Thirteen amino acids were characterised by a decrease in abundance consistent with extracellular NH_4_^+^ run-out. This trend was most striking for glutamine, asparagine, aspartic acid, isoleucine, and valine, which were all more than 10-fold depleted following N starvation, relative to N-replete conditions (0 h). In contrast, the abundance of four amino acids (arginine, lysine, proline, tyrosine) peaked following NH_4_^+^ starvation (5–7.5 h). This suggests that whilst N starvation is characterised by limitation in many amino acids, some pools are not only robust to the effects of starvation but may accumulate in this condition. This hypothesis is supported by the phenotype of the non-diazotrophic strains Δ*nifLA* and *nifK*_1__–__1203_, in which the same four amino acids accumulate in spite of the inability to recover from prolonged N starvation (Figure 6B). At 12.5 h, proline and lysine were approximately 10- and 5-fold more abundant, respectively, in Δ*nifLA* and *nifK*_1__–__1203_ cells relative to wild-type. In terms of absolute concentration, alanine, glutamate, and proline are in >5 mM excess in the non-diazotrophic strains. It is remarkable that most amino acids were at similar or moderately increased concentrations in both Δ*nifLA* and *nifK*_1__–__1203_, implying that prolonged N starvation is not characterised by a critical depletion of amino acids, even when there is the additional burden of Nif expression.

We calculated amino acid supply and demand in order to estimate the relative burden of Nif protein expression on the total substrate pool. Estimates for the production rate of each Nif protein (Supplementary Figure 12A) and the apparent demand for each amino acid substrate (Supplementary Figure 12B) were derived from the rate of change in measured protein concentration between 7.5 and 12.5 h in the wild-type and *nifK*_1__–__1203_. The observed rate of nitrogenase (NifHDK) synthesis during this period implies incorporation of >17,000 amino acid residues per second on average in the wild-type and approximately 950 per second in *nifK*_1__–__1203_. We do not account for Nif protein degradation in this model, such that amino acid demand may be underestimated. Furthermore, we approximated for how long the N starved amino acid pools (quantified at 5 h) could support Nif translation if they were finite in concentration and not replenished (Supplementary Figure 12C). Given the relative abundance and amino acid composition of the Nif proteins, alanine, glycine, glutamine, and leucine were in highest demand (>1000 molecules per second), and histidine and tryptophan in lowest demand (<250 molecules per second) in the wild-type. Taking into account pool concentrations, the least limiting amino acids were glutamate, proline, and alanine, which are sufficient to support >1 h of Nif protein expression in the wild-type and >10 h in *nifK*_1__–__1203_. However, 10 amino acid pools were insufficient to satisfy the theoretical demands of wild-type Nif protein translation for more than 5 min. These estimates support a model in which N fixation is necessary to support further Nif expression (see Figure 5C) and suggest that fixed N is rapidly re-distributed through amino acid metabolism *via de novo* biosynthesis and/or metabolism reflecting transamination. In the case of *nifK*_1__–__1203_, the approximately 10-fold lower levels of Nif protein accumulation may stem from rate limitation in a few amino acids, such as asparagine and histidine, which must be replenished by transamination alone.

### Neither GlnK or GlnB Is Strictly Necessary for the Onset of Diazotrophy

**FIGURE 7:**
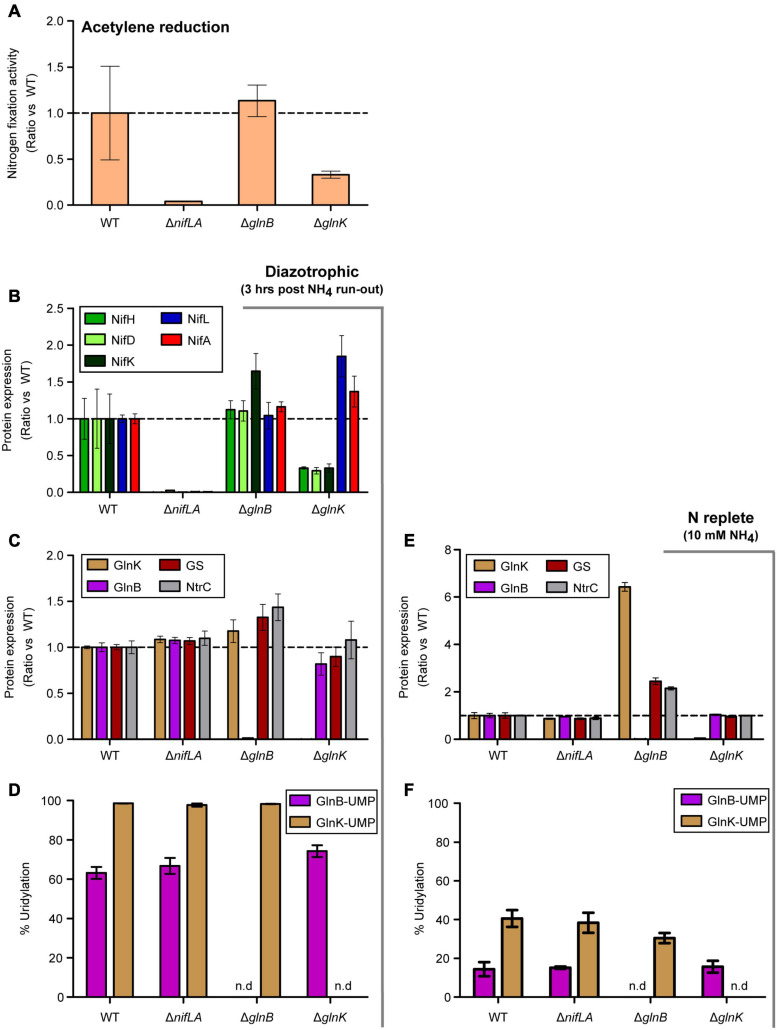
Effects of Δ*glnB* and Δ*glnK* knockout mutations on diazotrophy. *Ko* M5a1 wild-type, Δ*nifLA*, Δ*glnB*, and Δ*glnK* strains were cultured for 3 h following run-out of 0.5 mM NH_4_Cl in NFDM medium. Fold-change in various parameters with respect to wild-type: **(A)** Nitrogen fixation activity. Culture headspace was analyzed for ethylene evolution *via* the acetylene reduction assay. **(B)** Nif protein abundance. Extracted cell protein was supplemented with a mix of four QconCAT standards, trypsin digested, and the derived peptides analyzed *via* LC–MS. The absolute copy numbers of NifHDK (green shades) and NifLA (blue, red) were inferred from the sample/QconCAT peak area ratio for quantotypic peptides and QconCAT concentration, assuming an OD600 of 1.0 = 10^9^ CFU mL^–1^ and a cell volume of 10^–15^ L. **(C,E)** Ntr protein abundance. The absolute copy numbers of GlnK (brown), GlnB (pink), GS (maroon), and NtrC (gray). Dashed lines correspond to no fold change relative to wild-type. **(D,F)** PTM uridylylation status of PII-type proteins. Percentage uridylylation of GlnK (brown) and GlnB (pink) was inferred from the modified/total LC–MS peak area ratio for modifiable peptides. Note variable *y*-axis scales. Error bars represent SEM of three biological replicates. n.d. refers to no data shown.

To better understand the temporal signaling roles of the PII-type proteins GlnB and GlnK during diazotrophy, in particular the reported role of GlnK in activation of NifA, the effects of Δ*glnB* and Δ*glnK* knockouts on N fixation activity, Nif and Ntr protein expression and post-translational modification were assayed 3 h after NH_4_^+^ run-out. The Δ*glnK* mutant exhibited a threefold reduced acetylene reduction activity relative to wild-type, while Δ*glnB* had no notable effect (Figure 7A). Similar phenotypes were observed in the presence of 0.25 mM extracellular glutamine (equivalent N atoms to 0.5 mM NH_4_), which has been used interchangeably with NH_4_^+^ as a limiting N source (Supplementary Figure 13A). Consistent with acetylene reduction, NifHDK protein abundance was reduced threefold in Δ*glnK* cells but not in Δ*glnB* (Figure 7B). Albeit in a microtiter context rather than batch flask, which may lead to subtle differences in O_2_ concentration and redox state, diazotrophic growth was severely delayed in the Δ*glnK* mutant, whilst Δ*glnB* grows at the same rate as the wild-type (Supplementary Figure 13B). The knockout mutations were validated by the absence of independent signature peptides for GlnB and GlnK, respectively (Figures 7C,D). Given that non-zero levels of Nif abundance and nitrogenase activity were measured in both mutants, neither GlnK nor GlnB is strictly necessary for diazotrophy to occur in the presence of the remaining homologue. However, GlnK is more strongly influential in full de-repression of *nif* expression, consistent with nuanced roles for the partially redundant PII proteins. GlnB may exhibit some function in the dissociation of NifLA and de-repression.

Both NifLA abundance (Figure 7B) and the fraction of uridylylated GlnB (Figure 7D) were moderately increased in Δ*glnK*, suggesting that both N stress and NtrC activity were elevated. However, the fact that NifHDK were downregulated despite an increase in abundance of NifLA (Figure 7B) implies that the dominant role of GlnK is in dissociation of the NifLA complex and its role in perception of N stress for the activation of NtrC is of secondary importance. Whilst Δ*glnB* had no apparent effect on Nif expression, increased levels of GlnK, GS, and NtrC were observed in both diazotrophic and N-replete conditions (Figure 7C,E). Given that *glnK* expression is understood to be induced specifically under N limitation stress (as observed in Figure 5), it is notable that GlnK levels were elevated by more than sixfold in the Δ*glnB* strain, relative to wild-type, in the presence of excess NH_4_Cl (Figure 7E). This implies the absence of GlnB leads to perceived N limitation signaling despite actual N-replete conditions. In the context of GlnK expression as a marker for NtrC activity, the loss of the repressive effect of non-uridylylated GlnB on NtrB kinase activity may explain why NtrC phosphorylation, and therefore GlnK expression, is elevated in Δ*glnB*.

## Discussion

### Diazotrophy Involves Persistent N Stress Signaling

N run-out and upshift experiments enable faithful and non-intrusive study of the physiological adaptations induced in response to changes in environmental and cellular N status (Schumacher et al., 2013; Gosztolai et al., 2017). In contrast to other approaches used to establish N stress, such as rapid cell washing followed by medium replacement (Poza-Carrión et al., 2014) and continuous culture (Schreiber et al., 2016), N run-out enables both the pairwise comparison of distinct N statuses (N-replete and N starved) and the consumption-dependent kinetics of the intervening adaptive transition to be revealed. We performed NH_4_^+^ run-out experiments in which *Ko* M5a1 cells were sampled over a time course for multi-omics analyses of N-replete, N starved, and diazotrophic growth states. Nitrogen fixation activity was first detected within 2.5 h of complete NH_4_^+^ consumption, with evidence for diazotrophic growth observed at the subsequent time point (Figure 1). A notable hallmark of diazotrophy in these conditions was the continuation of N stress signaling despite the resumption of growth, with maximal levels of PII protein uridylylation reached prior to complete NH_4_^+^ starvation (at an extracellular NH_4_^+^ concentration approximately 100 μM) and remaining elevated for the remainder of the time course (Figure 5). This is perhaps not surprising given that glutamine, the key metabolic indicator of cellular N status, is consumed as the amino donor in the cyclical GS-GOGAT pathway necessary for assimilation of fixed NH_4_^+^ and decreases in concentration with NH_4_^+^ starvation (Figure 7).

The fact that expression of both nitrogenase and GS is dependent on NtrC activity implies that diazotrophic growth is inherently dependent on some level of N stress signaling. However, the accumulation of NtrC-dependent gene products (GlnK, GS) several hours prior to NifA-dependent *nif* gene products (Figure 5) suggests that the two regulons may be induced at different thresholds of N stress. The apparent delay in Nif expression may be due to differences in either NtrC-dependent promoter activities or the N stress thresholds at which post-translational events yield active NtrC and NifA. It is known that the *nifLA* promoter has a lower affinity for NtrC than the *glnA* promoter (Austin et al., 1987; Minchin et al., 1988), whilst the reported role for GlnK in liberating NifA from its inhibitive complex with NifL (Jack et al., 1999) may in principle occur at a different threshold N stress than GlnB/GlnK-dependent phosphorylative activation of NtrC.

### The Allocation of Resources to Nitrogenase Synthesis

Nitrogen fixation protein expression was induced rapidly alongside onset of N stress (Figure 5). Whilst the regulation of the *nif* genes is well understood (Dixon and Kahn, 2004), in particular their dependence on N stress and microaerobiosis (signalled *via* NifLA), this study reveals the scale with which gene expression capacity is redirected toward synthesis of nitrogenase during the diazotrophy transition, highlighted by the estimated fractions of the transcriptome and proteome accounting for *nif* transcripts and Nif proteins, respectively. According to our comparison of the *Ko* M5a1 transcriptome between N-replete and N-fixing conditions, the *nif* genes are among both the most highly upregulated and highly expressed sets of genes during the transition to diazotrophy (Figure 2). Here, the use of RNA-seq has the advantage over previous studies based on qRT-PCR techniques (e.g., Poza-Carrión et al., 2014) in that the relative expression of the *nif* genes as a function of the whole transcriptome can be determined qualitatively. Highly stringent regulation of the *nif* genes by NifA is reflected both by negligible transcription activation in N-replete conditions and the apparent absence of additional internal promoters or signatures of antisense regulation (Supplementary Figure 2). Putative σ^54^-dependent transcription start sites were identified for seven of the eight described *nif* operons, with the exception of *nifM*, although these remain hypothetical until followed up using a primary transcript selective methodology, such as differential RNA-seq (dRNA-seq).

The most notable feature of Nif protein expression dynamics was the accumulation of the structural nitrogenase subunits (NifHDK) at a higher rate than the accessory Nif proteins involved in biosynthesis and maturation (Figure 5C), mirroring the pattern observed in *Azotobacter vinelandii* (Poza-Carrión et al., 2014). This bifurcation likely reflects the optimal order and reaction rate of different protein functions during diazotrophy, with biosynthesis and maturation especially critical for early establishment while the concentration of nitrogenase may be more closely correlated to subsequent growth rate and fitness. Assuming a degree of subunit stability, once an optimal nitrogenase concentration is established, only minimal expression of the accessory Nif proteins that catalytically support maturation may be necessary. In *A. vinelandii*, despite being actively degraded during diazotrophic growth, NifB and NifEN provide sufficient FeMo-co for an excess of NifDK molecules as a result of multiple enzymatic turnovers (Martínez-Noël et al., 2011; Poza-Carrión et al., 2014). The pattern of Nif protein accumulation observed is also supportive of a simple model of polycistronic transcription of the *nif* operons, in line with our understanding of *nif* promoter architecture, with minimal post-transcriptional regulation. The stoichiometry of the proteins expressed from each operon remained relatively constant throughout the time course studied (Supplementary Figure 9) and the most upstream gene in each operon tended to be expressed at the highest protein copy, with the exception of NifE, which was consistently exceeded by the NifN concentration.

The concurrent measurements of absolute protein and intracellular amino acid concentrations performed in this study enable coarse grained predictions as to how intracellular N, both pre-existing and *de novo* fixed, is re-allocated to permit high levels of Nif expression in spite of N starvation conditions. The concentrations of most amino acids following N run-out would be insufficient as a finite substrate pool for the subsequent surge in Nif protein expression (Supplementary Figure 12), implying that their replenishment *via* a combination of *de novo* N fixation and scavenging of existing biomass is necessary. The fact that Nif proteins accumulated to only 10% of wild-type levels in the presence of a non-functional nitrogenase (*nifK*_1__–__1203_) provides strong evidence that fixed N is rapidly re-invested into further Nif expression. Focusing on the time window following the first detected N fixation activity (7.5–12.5 h), 900,000 excess Nif proteins were synthesised in wild-type relative to the non-diazotrophic *nifK*_1__–__1203_, requiring the supply of an additional 16,900 amino acids per second. The NifHDK concentrations at 10 h imply that there may be up to 35,000 nitrogenase enzymes per cell (70,745 NifK copies). Assuming a theoretical di-nitrogen reduction rate of 5 s^–1^ (Hageman and Burris, 1978) this equates to a total fixation rate in the range of 350,000 N atoms s^–1^, implying that it is indeed feasible for fixed N to account for the excess Nif protein expression, in addition to other housekeeping processes such as the onset of slow cell division between 10 and 12.5 h.

Whilst it is difficult to make robust comparisons across species boundaries, the general parameters of protein synthesis during diazotrophy can be inferred from earlier studies that have subjected bacterial models to growth under N limiting conditions. Li et al. (2018) observed a growth rate of *E. coli* on 1.9 mM NH_4_^+^ (0.1 doublings per hour) similar to that of diazotrophic *Ko* M5a1 in this study (0.098/h between 10 and 12.5 h). Assuming that total protein content in such a slowly growing cell equates to approximately 7.6 × 10^7^ polymerised amino acids, as measured by Bremer and Dennis (2008), it follows that Nif proteins account for around half of total cell protein following the onset of diazotrophic growth (52.2%; 12.5 h), with the nitrogenase largely responsible for this protein burden (46.3%). These estimates are somewhat higher than, but broadly in line with, previous measurements of Nif protein content (Dixon and Kahn, 2004). Under N-limiting conditions, active *E. coli* ribosomes are both relatively few in number (approximately 8000 copies per cell) and sparsely distributed along translating mRNA molecules (142 nt/ribosome) (Bremer and Dennis, 2008; Li et al., 2018). Considering the effects of both N limitation and growth at 25°C, it is probable that the translation elongation rate of *Ko* M5a1 ribosomes is constrained to five amino acids per second or less (Zhu et al., 2016; Li et al., 2018). If that assumption were to hold true then almost half (47.5%) of the active ribosome complement would need to be engaged in Nif protein translation to account for the accumulation rates observed between 7.5 and 12.5 h. Taken together, these rudimentary estimates imply that the proteome of early diazotrophy can be modelled as two compartments of broadly similar size, one consisting of the Nif proteins and one comprising all other proteins, with a reduced pool of active ribosomes divided between them. However, further studies involving both an accurate measurement of the total proteome size and ribosome profiling are necessary to test this hypothesis further.

### The Response of Amino Acid Pools to N Starvation and Diazotrophy

The reduced availability of functional ribosome general to N limited growth (Li et al., 2018) has been directly related to a decrease in the pool of amino acids used as substrates for translation. Amino acids are the common currency that couple metabolism, protein synthesis, and growth rate, with auto-regulatory mechanisms in place that tune ribosome concentration and translation rate to the amino acid supply and nutrient conditions (Scott et al., 2014). Sub-saturating levels of tRNA charging stimulates downregulation of ribosomal gene expression *via* the stringent response (Condon et al., 1995) along with ribosome hibernation (Sanchuki et al., 2017). Therefore, the decrease in abundance of many amino acids that occurs concurrently with extracellular NH_4_^+^ run-out (Figure 6 and Supplementary Data 3) may not only place a direct substrate limitation on translation rate but further constrain it indirectly *via* down-regulation of ribosome availability. Furthermore, these same regulatory mechanisms that couple amino acid supply to translation rate may function to explain why the N starved amino acid pools in non-diazotrophic mutants (Δ*nifLA* and *nifK*_1__–__1203_) remained so similar to wild-type despite no influx of fixed N. In effect, the active slowdown of translation rate acts to maintain a steady-state of amino acids and prevent complete run-out (Scott et al., 2014). Real-time measurement of translation elongation speed, as demonstrated by Zhu et al. (2016), in N starved and either diazotrophic or non-diazotrophic cell samples could be utilised in the future to test the hypothesis that translation rate increases in response to N fixation specifically.

The primary signal of cellular N status, glutamine, was depleted more than 25-fold during experimental NH_4_^+^ run-out (0–5 h). This is in line with previous reports in *E. coli* (Schumacher et al., 2013) and *Salmonella typhimurium* (Ikeda et al., 1996) and points to a model in which the perception of external nitrogen limitation *via* internal glutamine limitation is general to the *Enterobacteriaceae* family. Conversely, the fivefold decrease in glutamate concentration seen during the same time period contrasts with previous observations that this amino acid is relatively insensitive to NH_4_^+^ limitation (Brauer et al., 2006; Schumacher et al., 2013), even in *Klebsiella pneumoniae* specifically (Schmitz, 2000). Glutamate levels were partially restored by the 10 h time point, twofold higher that at 2.5 h. It is possible that variation in cell size throughout the diazotrophy transition may modestly skew calculations of amino acid concentrations which rely on an assumption of constant cell volume (1 fL). We have shown previously that the median cell volume of *E. coli* may decrease by approximately 30% following NH_4_^+^ run out (F. Heydenreich unpublished). Cell volume measurements were not performed in this study, however, a decrease in volume due to N starvation would effectively equate to an underestimation of intracellular metabolite concentrations.

Studying the non-diazotrophic Δ*nifLA* and *nifK*_1__–__1203_ mutants revealed several unexpected characteristics of amino acid metabolism during N starved conditions. As discussed above, prolonged N starvation did not lead to complete depletion of any one or more amino acids. Rather, in the absence of N fixation, subsets of amino acids accumulate to concentrations in excess of those observed in N-replete conditions (proline, lysine, and arginine) or in excess of time point-equivalent wild-type pools (glutamate, alanine, proline, lysine, and arginine). The accumulation of specific amino acids in non-diazotrophic mutants may signify bottlenecks in N metabolism when Nif expression is absent and/or nitrogenase function is lost. Low GS activity due to NH_4_^+^ limitation may explain the marginal excess of glutamate. Furthermore, both proline and arginine can be synthesised *de novo* from glutamate, which may explain their accumulation. Proline has also been shown to serve as an osmoprotectant during diazotrophy in *Ko* M5a1 (Le Rudulier et al., 1982). Alterations in glutamate metabolism cannot explain the accumulation of lysine, whose biosynthesis is unrelated. An alternative hypothesis is that some accumulating amino acids are derived preferentially from protein degradation and other N scavenging pathways.

### The Roles of the PII-Type Proteins in the Transition to Diazotrophy

Several studies have attempted to discern the distinct roles of the paralogous PII-type proteins GlnB and GlnK in the regulation of N metabolism. Functional redundancy between the pair is most apparent *in vitro*, whereby, for instance, uridylylated GlnK can activate both NtrB and the adenylation of GS and non-uridylylated GlnB can interact with the AmtB channel, albeit with lower affinity than the other paralogue in each case (Van Heeswijk et al., 1996; Atkinson and Ninfa, 1999; Javelle et al., 2004). Their distinct functional associations *in vivo* appear to relate as much to their condition-specific copy numbers as their structural differences, considering that GlnK exceeds constitutive GlnB many fold when upregulated during N limiting conditions. It has been proposed that GlnB represents the primary glutamine sensor in *E. coli*, with GlnK acting as a dynamic buffer to prevent excess N shock following starvation (van Heeswijk et al., 2013; Gosztolai et al., 2017).

The de-repression of NifA in a subset of diazotrophic bacteria such as *Ko* M5a1, however, is a PII-type function that has been attributed exclusively to GlnK (He et al., 1998; Jack et al., 1999), with GlnB only able to substitute for GlnK at excessively high and non-physiological concentrations (Arcondéguy et al., 1999). We observed that a Δ*glnK* knockout mutation reduced both the de-repressed level of nitrogenase activity and NifHDK protein levels by around two thirds when either NH_4_^+^ or glutamine were used as a limiting N source but still allowed N fixation at a reduced rate (Figure 7 and Supplementary Figure 13). This phenotype is in line with the 75% reduction in *nif* promoter activation seen previously in *K. pneumoniae*, albeit when using a strain in which *nifLA* were expressed independently of cellular N status from a low copy plasmid (Jack et al., 1999). However, it contrasts with the study by He et al. (1998), which reported complete repression of *nif* promoter activation in the absence of GlnK in a heterologous *E. coli* model system and founded the long-held view of an essential regulatory role. We propose that our study of GlnK in the native *Ko* M5a1 background is more representative of its true function during diazotrophy. Whilst the knockout strains generated in this report contain a *nptII* resistance cassette at the mutated loci, which may impose polar effects on neighbouring genes, it is important to note that previous studies have confirmed that the phenotypes of similarly derived Δ*glnK* strains are manifest independently of polar effects on the downstream *amtB* gene (Atkinson and Ninfa, 1998; He et al., 1998).

Although the Δ*glnB* mutation had no effect on N fixation or growth rate, our results suggest that GlnB is the more sensitive of the PII-type paralogues to changes in cellular N status. Whilst the GlnK uridylylation level remained at >90% following NH_4_^+^ runout, GlnB was marginally de-uridylylated following the onset of diazotrophic growth (Figure 5). Furthermore, the level of GlnB uridylylation varied in the range of 60–75% during N limiting conditions, suggesting that a trimer consisting of 2/3 uridylylated subunits is the dominant functional state. The contrasting uridylylation states of the two PII-type proteins may reflect different affinities for the UT/UR enzyme (GlnD) and allosteric signaling molecule 2-ketoglutarate (α-KG). It has been shown that GlnB is de-uridylylated around 10-fold faster than GlnK in the presence of moderate glutamine concentrations in *E. coli* (Atkinson and Ninfa, 1999), supporting the hypothesis that GlnB plays a rapid and fine-tuning role in cellular N signaling. The lack of a notable Δ*glnB* phenotype in the N limiting conditions studied may simply reflect the fact that GlnK is in excess, which enables it to substitute for GlnB but not to the same extent vice versa. In contrast, the Δ*glnB* mutant had a clear impact on NtrC activity in N-replete conditions when it represents the most abundant PII-type protein (Figure 7). Future studies might explore this hypothesis further by utilising a double Δ*glnBglnK* knockout as a baseline genotype in which to vary GlnB and GlnK expression levels independently. The fact that uncomplemented double Δ*glnBglnK* mutants render some diazotrophs unable to fix N (e.g., Michel-Reydellet and Kaminski, 1999) implies that whilst there may be redundancy between GlnB and GlnK, as observed here, some PII-type protein function is likely necessary for de-repression of *nif* gene expression. Our data would suggest that GlnB can function in place of GlnK, but at low constitutive copy number only minimal de-repression *via* NifLA is achieved.

### The Global Diazotrophic Transcriptome

Our comparison of the *Ko* M5a1 transcriptome between N-replete and N-fixing conditions revealed differential expression in more than 50% of genes (Figure 2 and Supplementary Figure 1), highlighting the breadth of cellular functions associated with both regulation of and adaptation to diazotrophic growth. As expected, many of the operons upregulated in diazotrophic conditions are associated with the conserved bacterial N stress response. Eighteen of the 25 canonical NtrC/Nac-regulated operons described in *E. coli via* microarray (Zimmer et al., 2000) were apparently upregulated here also. However, we also describe several putative NtrC-dependent operons relating to the metabolism of N-containing compounds (urea, nitrate/nitrite, purines) which may enable valuable scavenging functions during starvation conditions. There is potential value in performing a complete characterisation of the NtrC-dependent regulon in *Ko* M5a1, which may differ significantly to that of *E. coli* and provide a clearer picture of how N fixation is associated to other modules of N metabolism. Whilst comparative genomic analyses indicate a high degree of similarity between *K. pneumoniae* and *E. coli*, almost 800 genes are unique between the two species, many of which are thought to correspond to novel metabolic phenotypes and nutrient growth capabilities (Liao et al., 2011).

The gene families apparently downregulated during diazotrophy include several associated with gene expression and cell division, suggesting that the slow growth rate observed during N starvation is in part regulated. The alarmone of the stringent response, ppGpp, is released by the ribosome-associated ppGpp synthetase (RelA) and in *E. coli* the expression of *relA* is induced by NtrC during N stress (Brown et al., 2014). This study saw upregulation of *relA* in diazotrophic cells, suggesting that the stringent response may be responsible for a large component of differential global gene expression, downstream of NtrC, under N starved conditions. The stringent response has also been strongly implicated in the global deceleration and hibernation of ribosomes (Li et al., 2018).

### Concluding Remarks

Given the longstanding biotechnological interest in enhancing biological nitrogen fixation, it is remarkable that our understanding of diazotrophic bacteria has seldom progressed beyond a description of the various gene regulatory mechanisms governing *nif* gene expression to complete systems-level studies that integrate omics datasets and their interactions. Various refactored and synthetically inducible *Ko* M5a1 *nif* gene clusters reproduce only a fraction of wild-type nitrogenase activity in heterologous hosts (Temme et al., 2012; Ryu et al., 2020), highlighting the fact that even the most cutting edge of synthetic biology tools cannot compensate for an incomplete predictive model of native physiology. The genetic organization of *nif* genes would suggest that the N fixation function is sufficiently modular to be readily transferred between chassis microorganisms (Dixon and Postgate, 1972). However, it is deeply embedded in the context of multiple aspects of physiology beyond N status, including redox state, energy level, oxygen status, and metallochemistry.

This study provides a methodological framework within which predictive models of free-living diazotrophs can be developed and we have generated several hypotheses to be further explored. Analyses of Nif expression at the transcript and protein levels reveal a switch-like transition from tightly repressed to highly upregulated in response to N status. Given the scale of cellular burden associated with Nif expression, it is critical that metabolic engineering approaches applied to diazotrophy incorporate elements of temporal control in response to multiple stimuli.

Targeted mass spectrometry for absolute protein quantification represents an improvement in accuracy and sensitivity over previous methods used to estimate Nif protein levels, such as immunoblotting alongside external purified standards for semi-quantitative estimation of Nif expression in *A. vinelandii* (Poza-Carrión et al., 2014). Mass spectrometry capability is becoming ever more accessible and the QconCAT approach to method development, in particular, enables multiplexed, high-throughput, and reproducible protein quantification. Digested peptide samples can be further assessed *via* untargeted proteomics. In the context of our observation that Nif proteins make up a significant fraction of the diazotrophic proteome, a critical objective of further studies should be to assess the most significant, and perhaps compensatory, changes that occur in the general non-Nif compartment of the proteome. Likewise, global proteomics may shed light on the Δ*nifLA* and *nifK*_1__–__1203_ phenotypes, in particular with regard to whether the excess of free amino acids is reflected by compensatory changes in non-Nif protein levels.

We also show that co-extraction of proteins and amino acids can shed light on the availability and allocation rate of N resources prior to and during starvation. However, a limitation of this study is that it is not possible to distinguish directly which N atoms in cellular resource pools, such as amino acids, are derived *de novo* from N_2_ fixation or from pre-existing biomass *via* degradation and scavenging. Utilizing stable N isotopes (^15^N) to differentially label atmospheric N_2_ and N-containing biomass would address this question while being well suited to a mass spectrometry approach.

Finally, in light of growing evidence that phenotypic heterogeneity is a pervasive fitness strategy for many biotechnologically relevant microbes, even within controlled, artificial environments (Binder et al., 2017), it is important to note that many of the methods employed to study the regulation of diazotrophy, as exemplified here, assume homogenous cell behavior and that bulk measurements therefore represent the average cell in the population. Indeed, single-cell studies performed by Schreiber et al. (2016) determined that *Ko* M5a1 exhibits mixed-substrate growth with distinct N_2_-fixing and NH_4_-feeding sub-populations when grown continuously in states of intermediate N stress. However, the fact that diazotrophy was the homogenous phenotype under complete NH_4_^+^ starvation would imply that this behavior can be assumed following complete NH_4_^+^ run-out in this study.

## Data Availability Statement

The datasets presented in this study can be found in online repositories. The names of the repository/repositories and accession number(s) can be found in the article/Supplementary Material.

## Author Contributions

CW, JS, and MBu designed the study. CW, AL, and CH performed the experiments. MBe contributed to analytical chemistry (LC–MS). IK-L and MC contributed to the analysis of transcriptome data. CW analyzed the data and prepared the manuscript. All authors contributed to critical review and editing of the manuscript.

## Conflict of Interest

The authors declare that the research was conducted in the absence of any commercial or financial relationships that could be construed as a potential conflict of interest.

## Publisher’s Note

All claims expressed in this article are solely those of the authors and do not necessarily represent those of their affiliated organizations, or those of the publisher, the editors and the reviewers. Any product that may be evaluated in this article, or claim that may be made by its manufacturer, is not guaranteed or endorsed by the publisher.
