# Supplementary material for: Resource Allocation During the Transition to Diazotrophy in Klebsiella oxytoca
Source: Front Microbiol. 2021 Aug 9;12:718487. doi: 10.3389/fmicb.2021.718487 (PMC8381380; doi:10.3389/fmicb.2021.718487)
Supplement: Supplementary file 4 [file Data_Sheet_1.pdf]

Supplementary Material

1 Supplementary Tables

Supplementary Table 1. Strains, plasmids and oligonucleotides used in this work.

**Strain**	**Genotype**	**Reference or Source**
E. coli		
BL21(DE3) Δ*lys*Δ*arg*	Derivative of Novagen BL21(DE3). F^{-}, *dcm*, *ompT*, *lys*, *arg*, *hsdS*(r_B^- m_B^-), *gal* λ(DE3)	Matic et al., 2011
K. oxytoca		
M5a1	Nif^+, Amp^r	Ruth Schmitz (Christian Albrechts University, Kiel)
Δ*nifLA*	M5a1(Δ*nifLA*::*nptII*). Amp^r Kan^r	This study
nifK$_{1\text{-}1203}$	M5a1(*nifK*$_{\Delta 1204\text{-}1545}$::*nptII*). Amp^r Kan^r	This study
Δ*glnB*	M5a1(Δ*glnB*::*nptII*). Amp^r Kan^r	This study
Δ*glnK*	M5a1(Δ*glnK*::*nptII*). Amp^r Kan^r	This study
**Plasmid**	**Description**	**Reference or Source**
pGEM-T-KanFRT	pGEM-T derivative containing a *nptII*-FRT selection marker cassette. Amp^r Kan^r	Zumaquero et al., 2010
pKD46	Lambda Red recombinase expression plasmid carrying nucleotides 31088-33241 of phage Lambda (*gam*, *bet*, *exo*) under the control of P*ara*. Temperature	Datsenko and Wanner, 2000

	sensitive replication (repA101ts). Amp^r. GenBank accession: AY048746	
pET100/D-TOPO-QNif1	Life Technologies pET100/D-TOPO expression vector carrying the coding gene for protein standard QNif1 in-frame with N-terminal 6-His tag. Amp^r	This study
pET100/D-TOPO-QNif2	Life Technologies pET100/D-TOPO expression vector carrying the coding gene for protein standard QNif2 in-frame with N-terminal 6-His tag. Amp^r	This study
pET100/D-TOPO-QNif3	Life Technologies pET100/D-TOPO expression vector carrying the coding gene for protein standard QNif3 in-frame with N-terminal 6-His tag. Amp^r	This study
pET100/D-TOPO-QNtr	Life Technologies pET100/D-TOPO expression vector carrying the coding gene for protein standard QNtr in-frame with N-terminal 6-His tag. Amp^r	This study
**Oligonucleotide**	**Sequence** (binding region in upper case)	**Target**
nifLA_mutF	ccgcggcgtccctgtcacggtgtcggacaaattgtcataactgcgacacaggagtttgcg GTGTAGGCTGGAGCTGCTTC	pGEM-T-KanFRT
nifLA_mutR	acaaattgtcgcaattccgccgcgctggcgacaatgtcctgaatctcacataaggcttcaT CCTCCTTAGTTCCTATTCCG	pGEM-T-KanFRT
nifK_mutF	gagctgggctgcgagccaacggtgatcctgagccataacgccaacaaacgctggcaaa aaGTGTAGGCTGGAGCTGCTTC	pGEM-T-KanFRT
nifK_mutR	catactccctcctggccccgcatgacgcggggcacctgatggttaacggacgagatcga aTCCTCCTTAGTTCCTATTCCG	pGEM-T-KanFRT
glnK_mutF	ctgcggccattaccgaattctgactggaggggacttatgaagctggttaccgtggtaatcG TGTAGGCTGGAGCTGCTTC	pGEM-T-KanFRT
glnK_mutR	tgttttcattgttgctatcttcattgtttcgttccccatcactgtgtggtctgaaagttaTCCT CCTTAGTTCCTATTCCG	pGEM-T-KanFRT
glnB_mutF	taggatgacgcaagttatgatacggttagcaaccggttacgcgactaagacaggaacacc GTGTAGGCTGGAGCTGCTTC	pGEM-T-KanFRT

glnB_mutR	cggggctacaaatctggatggaattgaagttcttaagccatcatccgtcttttaagcttaTCCTCCTTAGTTCCTATTCCG	pGEM-T-KanFRT

Notes: Amp^r, ampicillin resistant; Kan^r, kanamycin resistant.

2 Supplementary Figures

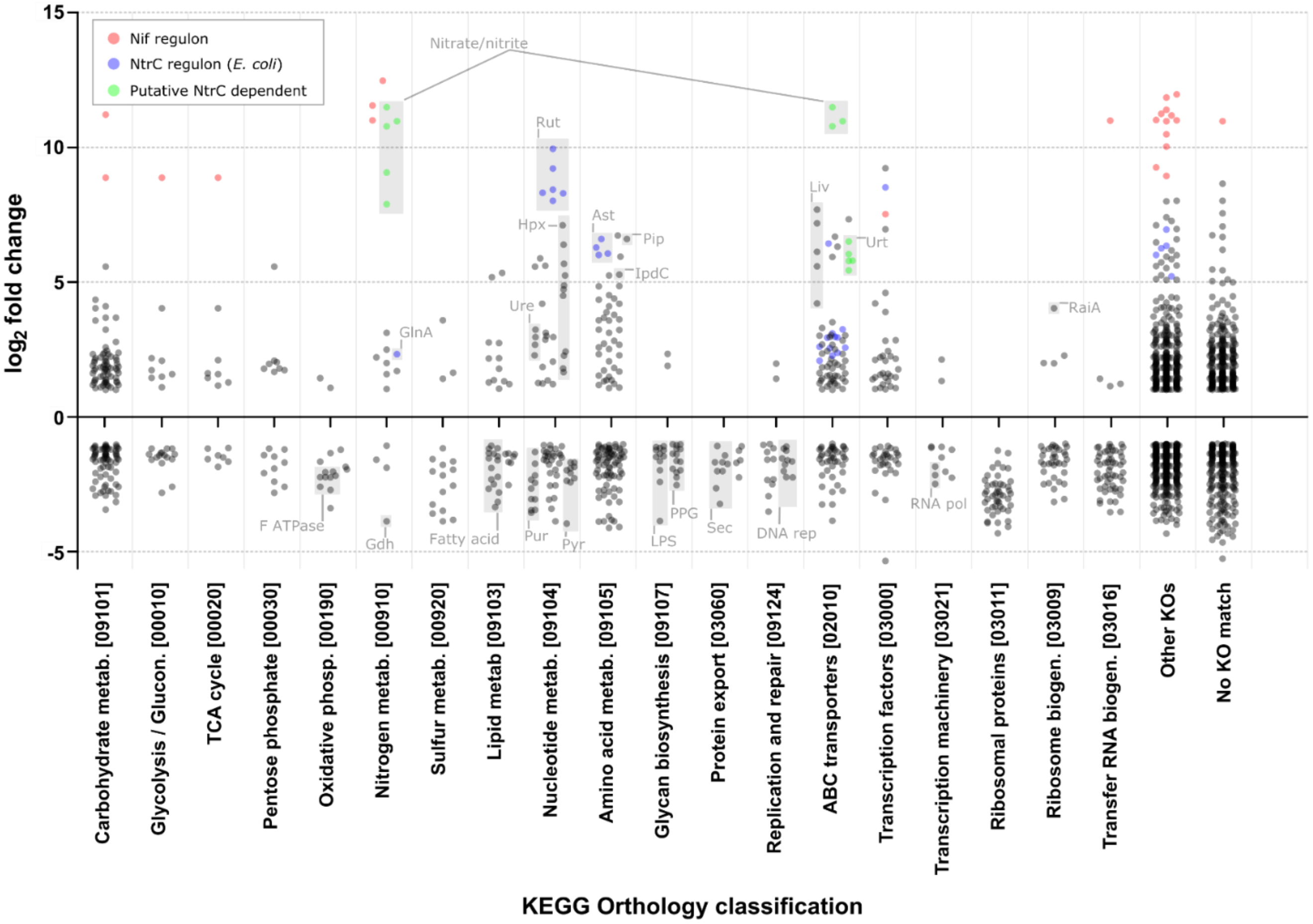

Supplementary Figure 1. Functional classification of genes differentially expressed during diazotrophy. Log_2 fold change of genes differentially expressed (DEGs) by more than 2-fold in N-fixing conditions (0.5 mM NH_4Cl run-out) with respect to N replete (10 mM NH_4Cl) conditions. DEGs are grouped according to relevant KEGG Orthology (KO) classifications, assigned to translated protein sequences using the KEGG Automatic Annotation Server. Some DEGs are classified by more than one KO. DEGs classified by KOs not listed explicitly are grouped under Other KOs. DEGs belonging to the known *nif* and NtrC-dependent regulons are highlighted in red and blue, respectively. Other highly-upregulated DEGs with putative sigma-54 promoters are highlighted in green. Grey boxes and annotations are given for some specific DEG groups. Abbreviations: Ast, arginine succinyltransferase; Gdh, Glutamate dehydrogenase; GlnA, glutamine synthetase; IpdC, Indole-3-pyruvate decarboxylase; Liv, leucine isoleucine valine; LPS, Lipopolysaccharide; Pip, proline iminopeptidase; pol, polymerase; PPG, Peptidoglycan; Pur, purine nucleotide biosynthetic; Pyr, pyrimidine nucleotide biosynthetic; RaiA, ribosome-associated inhibitor A; rep, replication; Rut, pyrimidine utilization; Urt, urea import.

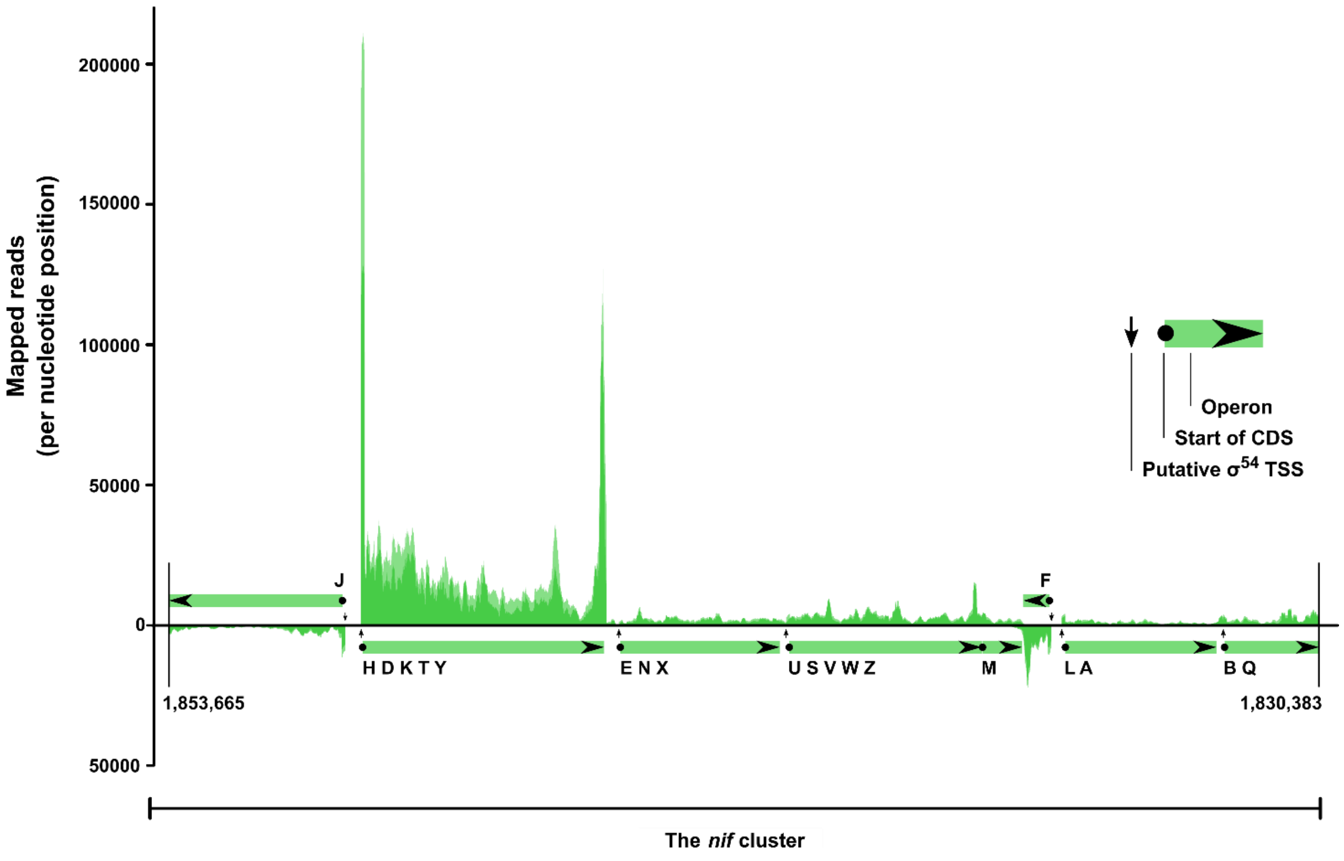

Supplementary Figure 2. RNA-seq read mapping to the *nif* gene cluster. The 20 *nif* genes are arranged according to 8 discrete transcriptional units, with *nifF* and *nifJ* on the reverse strand relative to *nifHDK*. Green bars delineate the coding sequences of each operon/gene, with circles and arrows representing the 5' translational start site and the reading direction, respectively. Mapping data corresponding to the transcriptome sampled in N-fixing conditions is represented by read density per nucleotide position. Two biological replicates are represented by two shades of green. Putative sigma-54 dependent TSSs at the 5' end of each transcriptional unit are signified by small vertical arrows.

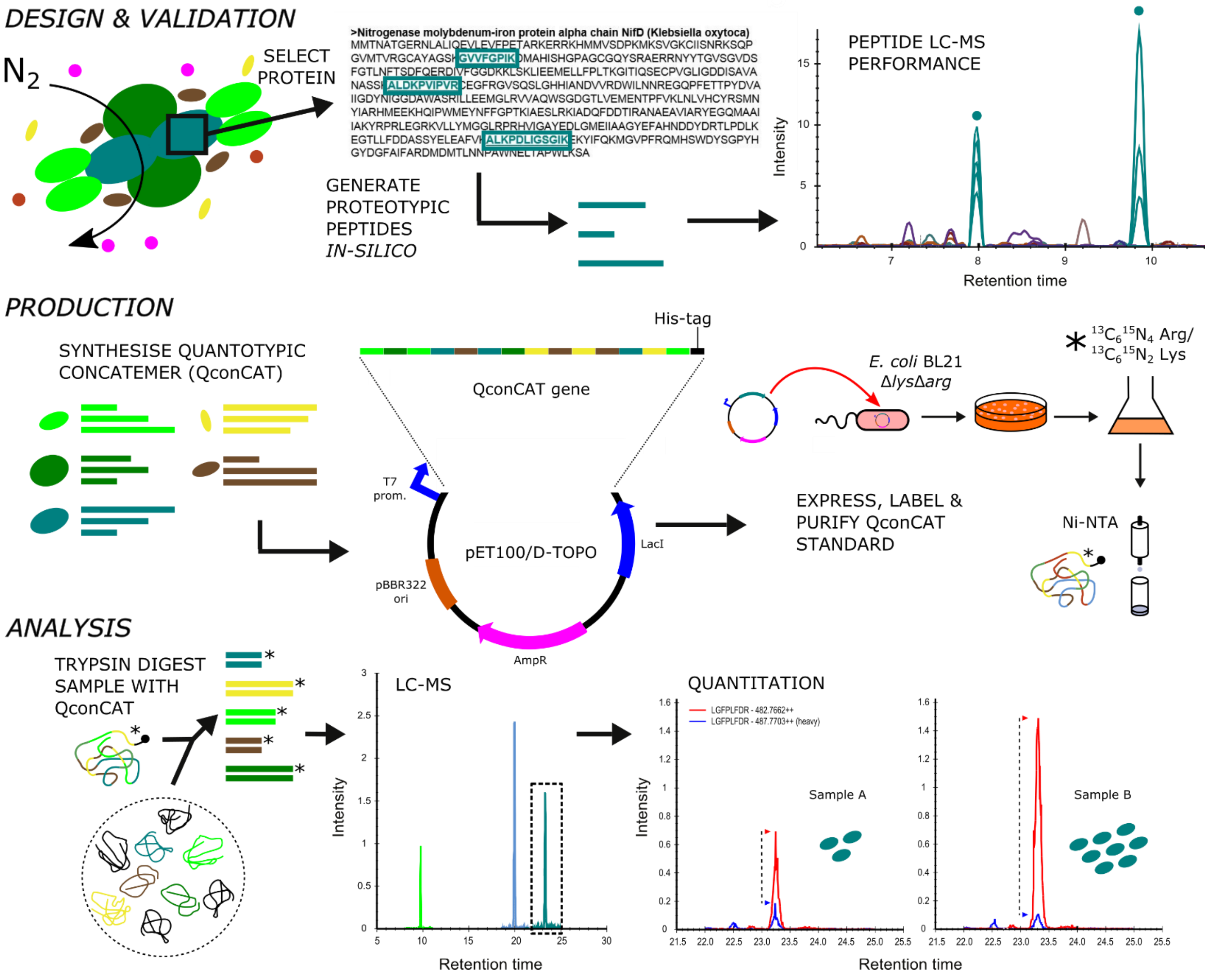

Supplementary Figure 3. Summary of QconCAT methodology applied to Nif protein quantification. Design and validation: Nif protein sequences are subject to *in silico* analysis for selection of tryptic peptides. Candidate peptide transitions are assimilated into selected reaction monitoring (SRM) methods and their presence and performance in biological samples assessed empirically via LC-MS. Production: Validated peptides from multiple target Nif proteins are concatenated to yield a single QconCAT protein sequence. The synthetic QconCAT gene is encoded on an expression plasmid and transformed into an *E. coli* protein expression strain for *in vivo* labelling in the presence of heavy ^{13}C ^{15}N amino acids. The His-tagged QconCAT protein is purified from insoluble cell protein extract via nickel-affinity chromatography. Analysis: A known concentration of QconCAT is added to biological protein samples prior to trypsin digest, which yields ‘light’ sample and ‘heavy’ standard isomers for each target peptide. The light/heavy LC-MS peak area ratio is used for accurate protein quantitation.

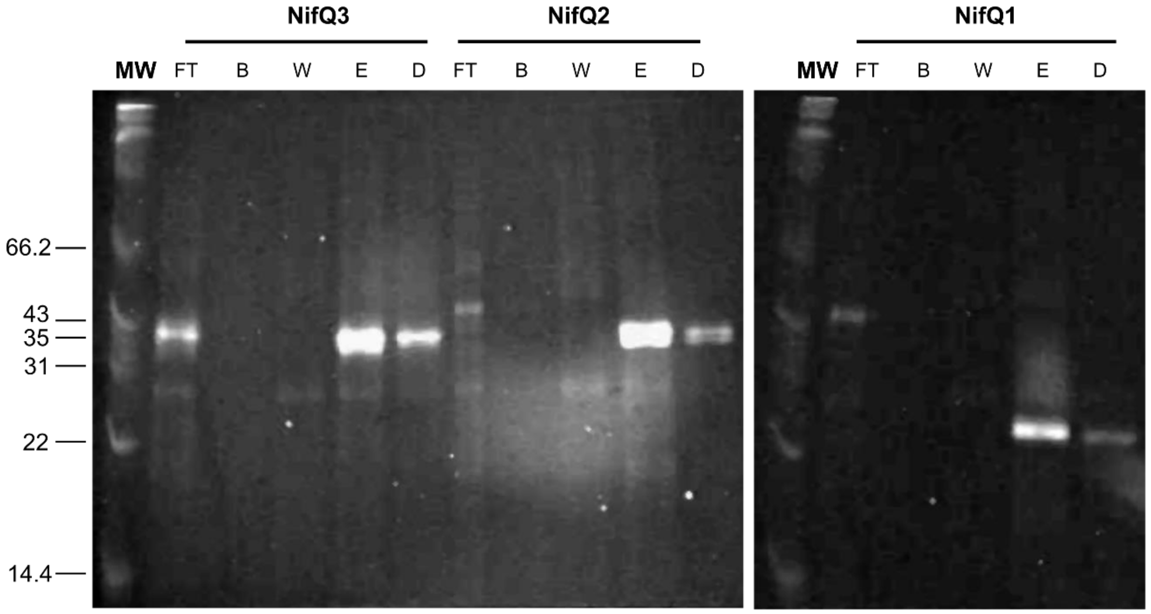

Supplementary Figure 4. Purification of Nif QconCAT standard proteins. 15% polyacrylamide gel showing samples of QconCAT protein sampled at consecutive steps of Ni-affinity purification: molecular weight marker (MW); column loading flow-through (FT); binding step (B); wash step (W); elution (E); post-dialysis (D). Expected protein molecular weights: QNif1, 29.7 kDa; Nif2, 37.6 kDa; QNif3, 36.4 kDa.

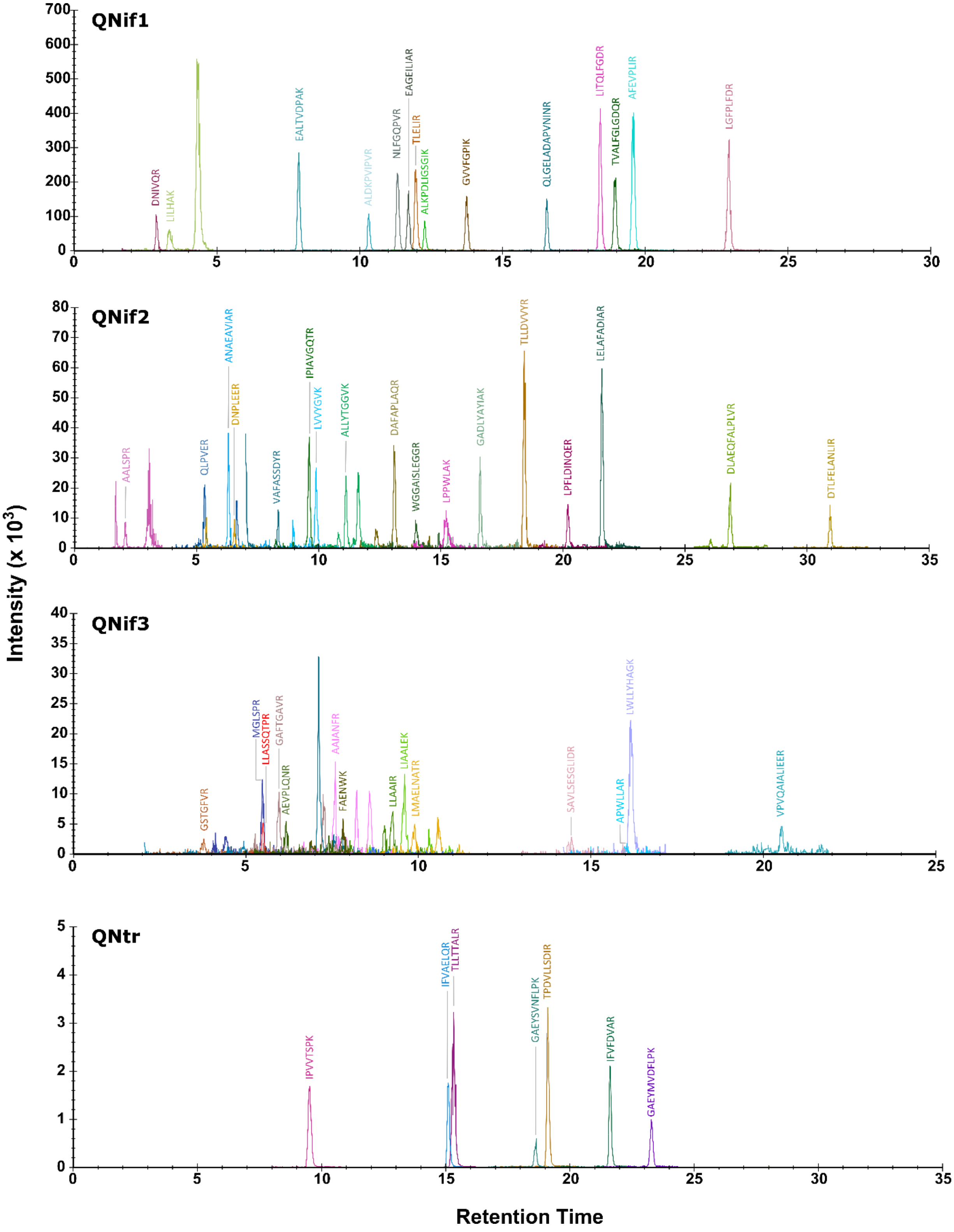

Supplementary Figure 5. LC-MS chromatograms of trypsin-digested QconCATs. Peaks are annotated with corresponding peptide sequences. Peak area shown is representative of summed selected reaction monitoring (SRM) transitions, constrained by 3 min retention time windows.

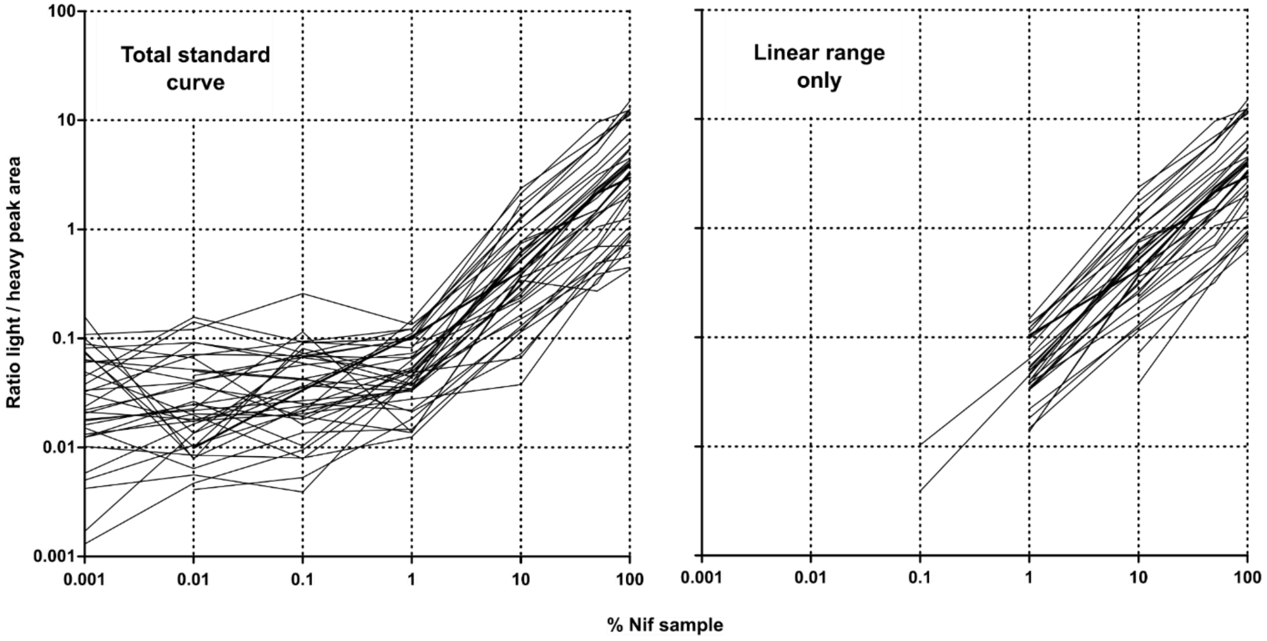

Supplementary Figure 6. Validation of peptide linearity. 10-fold dilution series of sample protein (wild-type, diazotrophic) supplemented with a constant concentration of QconCATs. Sample protein concentration and the sample/QconCAT LC-MS peak area ratio are plotted on a log10 scale. Left panel: all data points. Right panel: non-linear data points removed. Peptides exhibiting poor linearity were removed from the final SRM method.

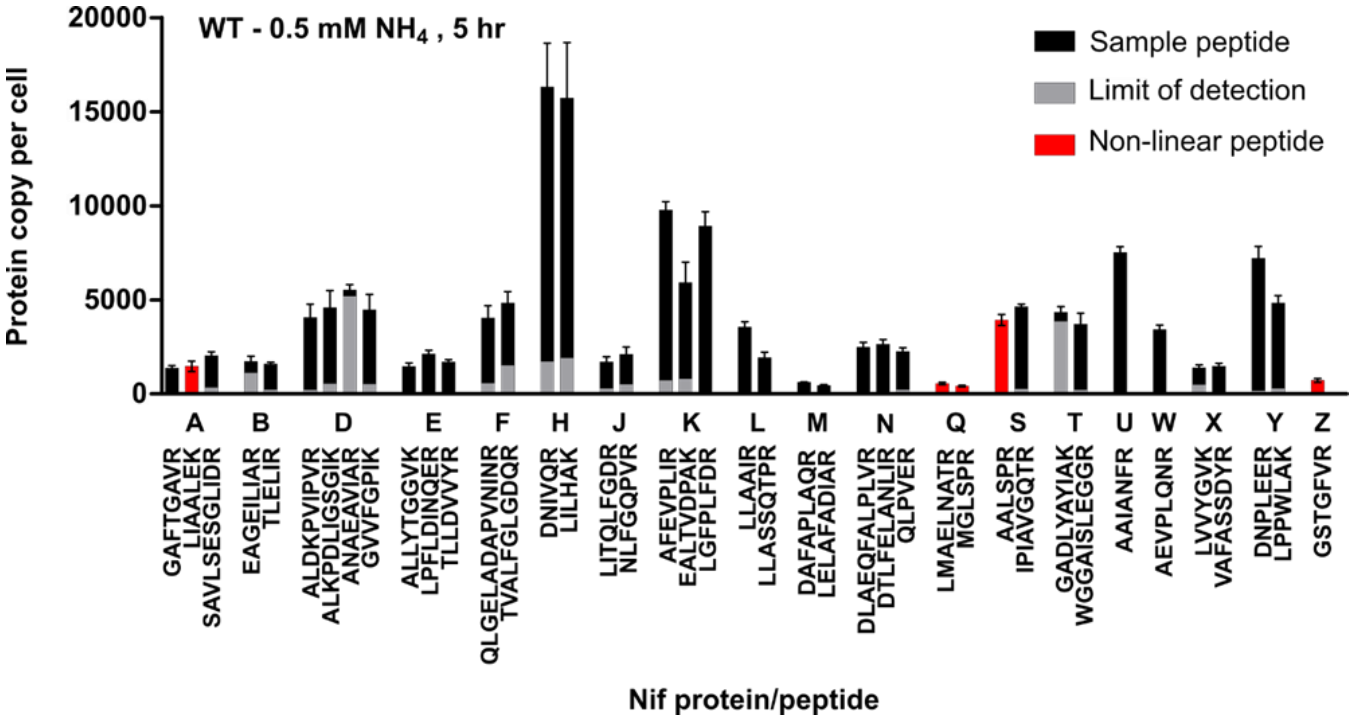

Supplementary Figure 7. Validation of Nif protein quantitation by peptide. Absolute copy number of Nif proteins in a diazotrophic cell extract inferred by the sample/QconCAT peak area ratio of specific peptides. Copy number estimates assume an OD_{600} of 1.0 = 10^9 CFU ml^{-1} and a cell volume of 10^{-15} L. Error bars represent SEM of 3 biological replicates. Limit of detection (overlaid in grey) is inferred via linearity of peptide response (see **Supplementary Figure 5**). Peptides highlighted in red show no linearity and are excluded from the final SRM method.

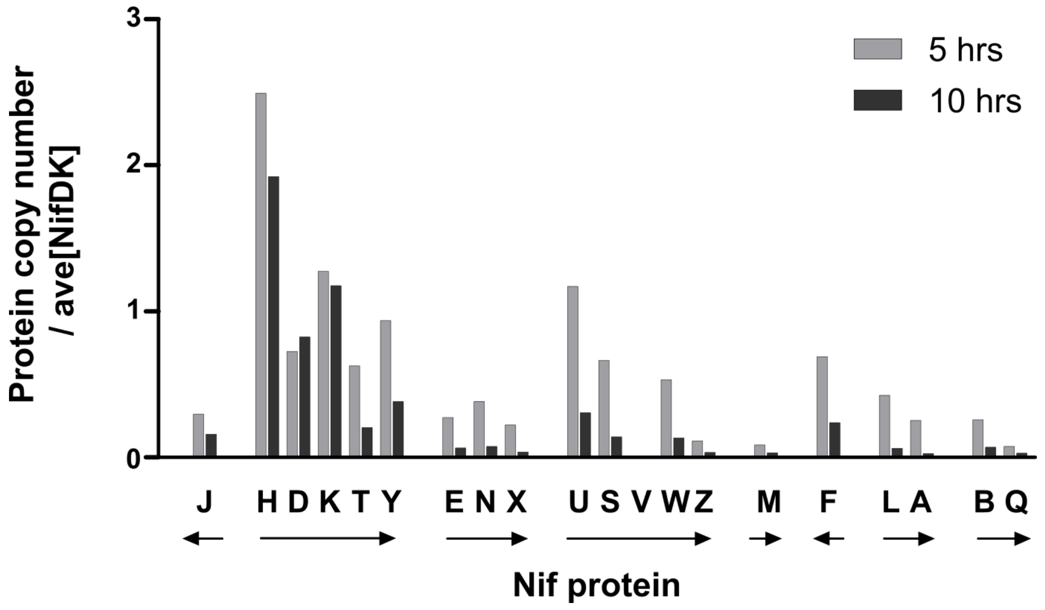

Supplementary Figure 8. Nif protein stoichiometry relative to the nitrogenase MoFe protein. Ratio of absolute Nif protein copy number to the average of NifDK in wild-type cells at 5 (light grey bars) and 10 hours (dark grey bars) following supplementation with 0.5 mM NH_4Cl. Absolute copy number inferred from the sample/QconCAT LC-MS peak area ratio for quantotypic peptides and QconCAT concentration, assuming an OD600 of 1.0 = 10^9 CFU ml^{-1} and a cell volume of 10^{-15} L. Nif proteins are displayed according to genomic organization with arrows representative of transcriptional units.

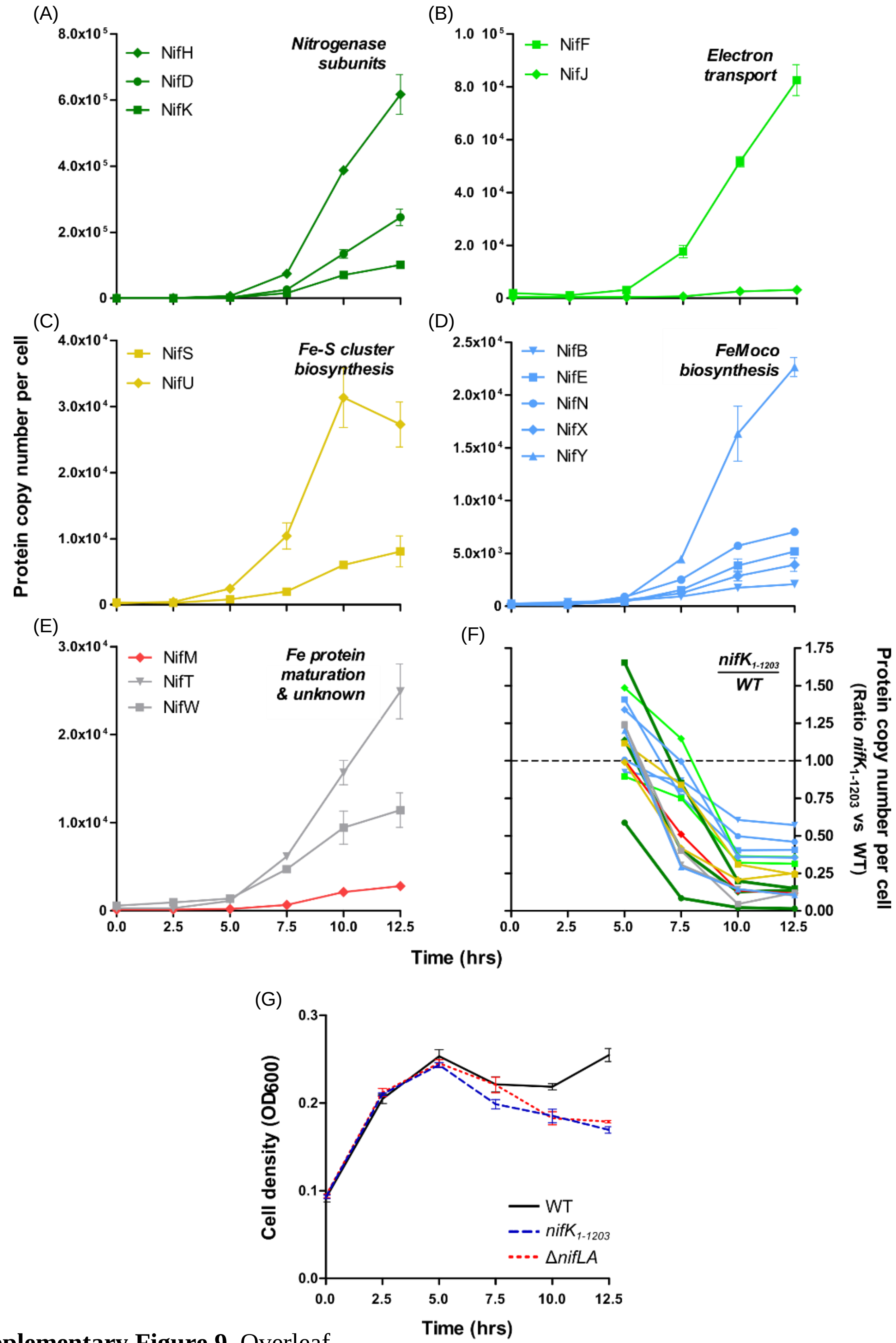

Supplementary Figure 9. Overleaf.

Supplementary Figure 9. Dynamics of the Nif protein accumulation during the diazotrophy transition. M5a1 wild-type was cultured for 12.5 hours in NFDM medium supplemented with 0.5 mM NH_4Cl under micro-aerobic conditions. Cell protein was extracted at 2.5-hour intervals, supplemented with a mix of QconCAT standards and trypsin digested. Absolute copy numbers of 15 Nif proteins were inferred from the sample/QconCAT LC-MS peak area ratio for quantotypic peptides and QconCAT concentration, assuming an OD600 of 1.0 = 10^9 CFU ml^{-1} and a cell volume of 10^{-15} L. Error bars represent SEM of 3 biological replicates. Nif proteins organized into inset panels according to functional group: **(A)** Nitrogenase; **(B)** electron transport; **(C)** Fe-S cluster biosynthesis; **(D)** FeMoCo biosynthesis; **(E)** Fe protein maturation and unknown. **(F)** Ratios of Nif protein copy number in $nifK_{1\text{-}1203}$ compared to wild-type between 5-12.5 hours. Ratio of means of 3 biological replicates. Annotations as in panels A-E. **(G)** Cell density (OD_{600}) of wild-type (black line), $nifK_{1\text{-}1203}$ (blue, dashed lines) and Δ*nifLA* (red, dotted line) strains measured immediately prior to sampling of protein.

(A)

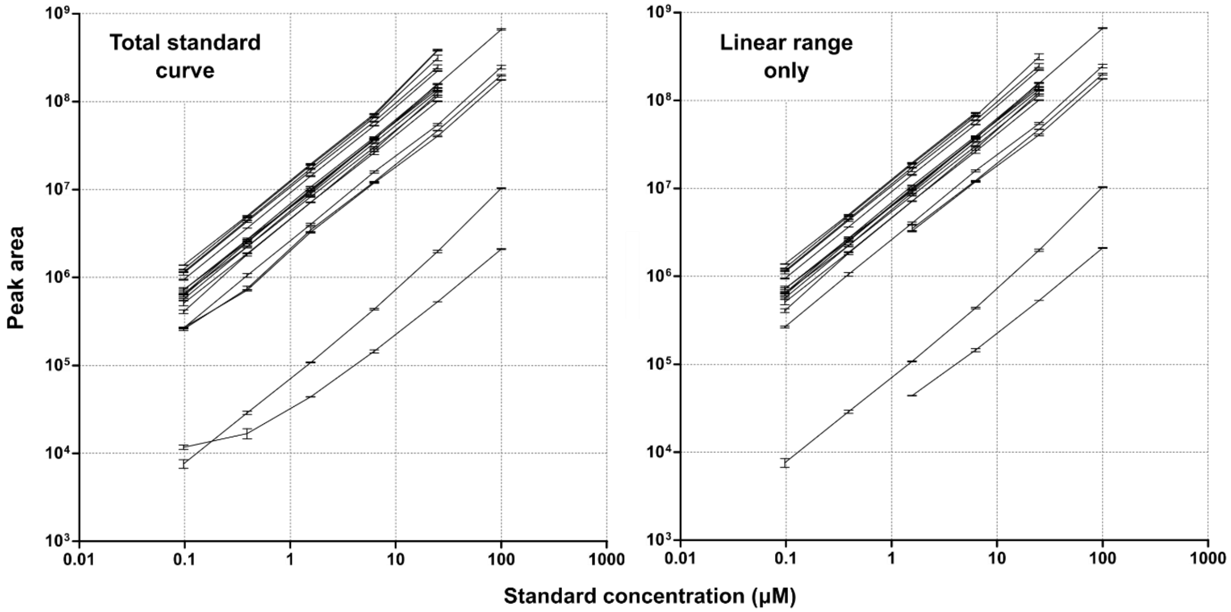

(B)

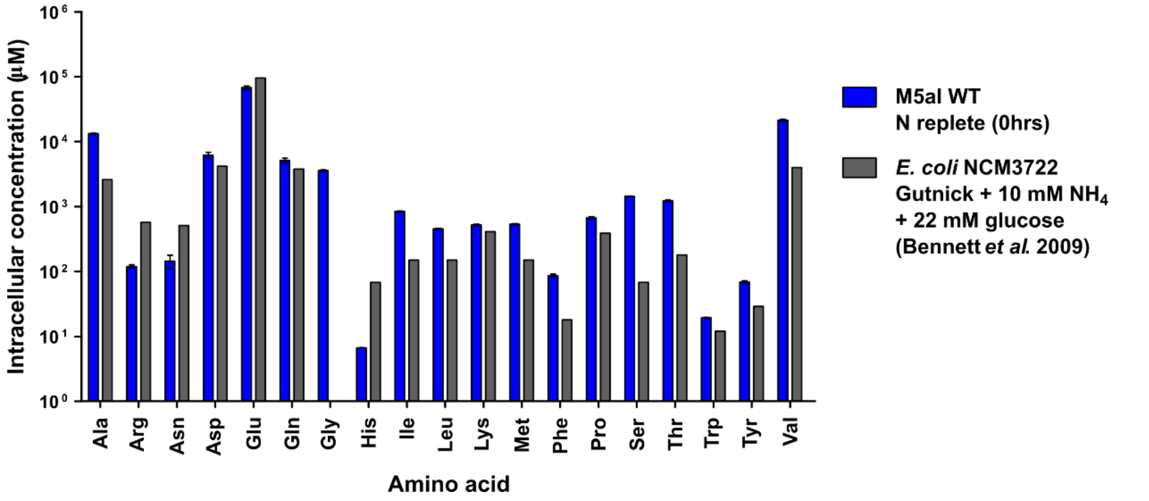

Supplementary Figure 10. Absolute quantitation of intracellular amino acid pools. **(A)** Validation of amino acid standard curve linearity. LC-MS peak area for a 4-fold dilution series of amino acids between 100-0.1 µM plotted on a log10 scale. Left panel: all data points. Right panel: non-linear data points removed. **(B)** Validation of amino concentration. Estimates of intracellular pool concentrations in M5aI wild-type cultured in N replete medium (blue) compared to published concentrations for *E. coli* K-12 strain NCM3722 cells cultured under similar conditions (grey) (Bennet et al 2009; media composition: 4.7g/L KH_2PO_4, 13.5g/L K_2HPO_4, 1 g/L K_2SO_4, 0.1 g/L $MgSO_4 \cdot 7H_2O$, 10 mM NH_4Cl, 4g/L glucose). Polar metabolites were derivatized with the AccQTag reagent (6-aminoquinolyl-N-hydroxysuccinimidyl carbamate) and analysed via LC-MS. Absolute amino acid concentrations were calculated by referencing external standard curves, assuming a cell volume of 10^{-15} L. Note log10 scale.

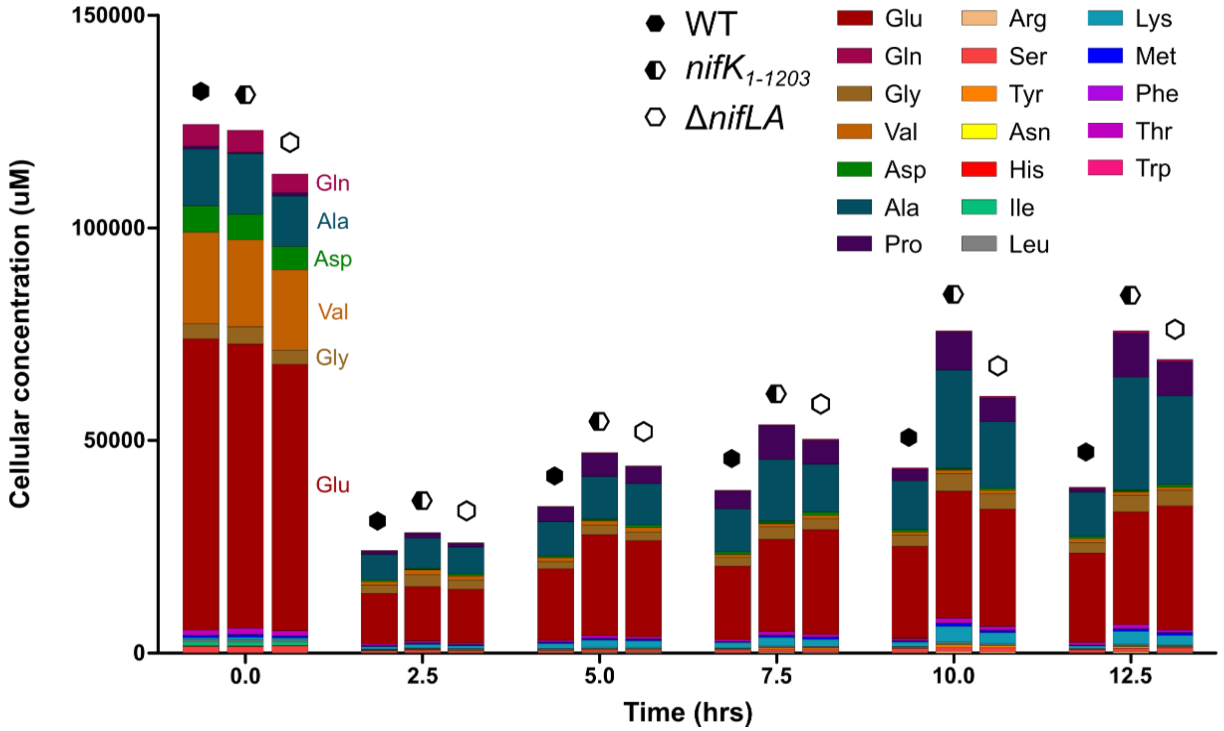

Supplementary Figure 11. Time-course of intracellular amino acid pool concentrations. M5a1 wild-type (full hexagon), *nifK*$_{1\text{-}1203}$ (half hexagon) and Δ*nifLA* (empty hexagon) strains were cultured for 12.5 hours in NFDM medium supplemented with 0.5 mM NH_4Cl. Polar metabolites were extracted at 2.5 hour intervals, derivatized with the AccQTag reagent (6-aminoquinolyl-N-hydroxysuccinimidyl carbamate) and analysed via LC-MS. Absolute amino acid concentrations were calculated by referencing external standard curves, assuming a cell volume of 10^{-15} L. The mean concentration of total intracellular amino acids across 3 biological replicates is shown, with specific amino acids annotated by color.

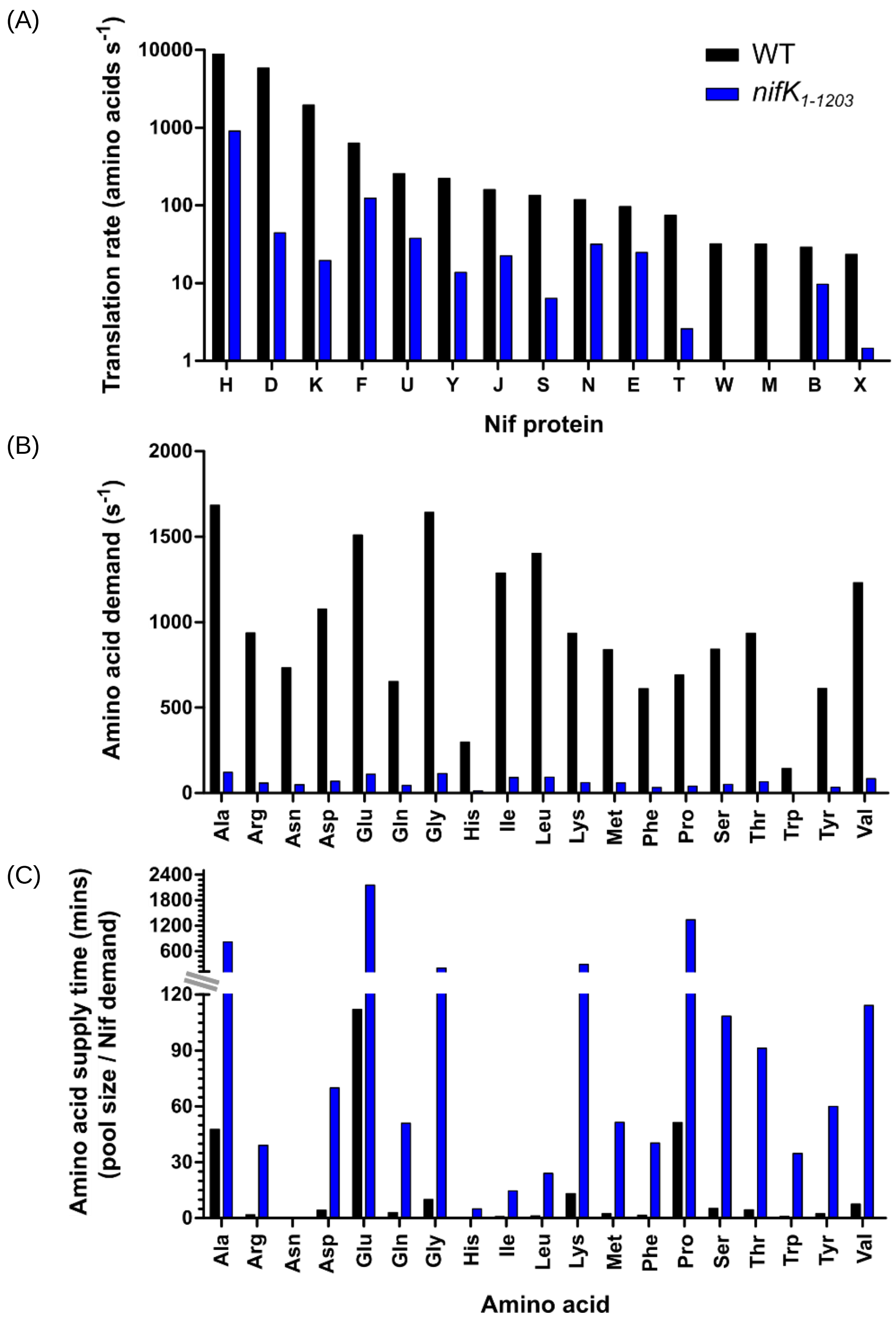

Supplementary Figure 12. Model of amino acid consumption by Nif protein synthesis in wild-type (black) and *nifK*1-1203 (blue). **(A)** Rate of amino acid consumption for each Nif protein per second. The change in absolute copy number between two discrete sampling time points (see **Figure 5c**; 7.5-12.5 hours) was used to calculate an approximate steady-state rate of Nif protein expression. Data is shown on a $\log_{10}$ scale. **(B)** Estimated amino acid demand. Per-second consumption of each amino acid for proxy steady-state Nif protein synthesis. Amino acid composition was determined using database sequences (GenBank WGS accession JAFHKG010000000). **(C)** Estimated amino acid supply time for Nif protein synthesis, assuming intracellular pool sizes reflect the onset of N starvation (see **Supplementary Data 3**; 5 hours) and are fixed and finite. Data is shown on two distinct y-axis scales.

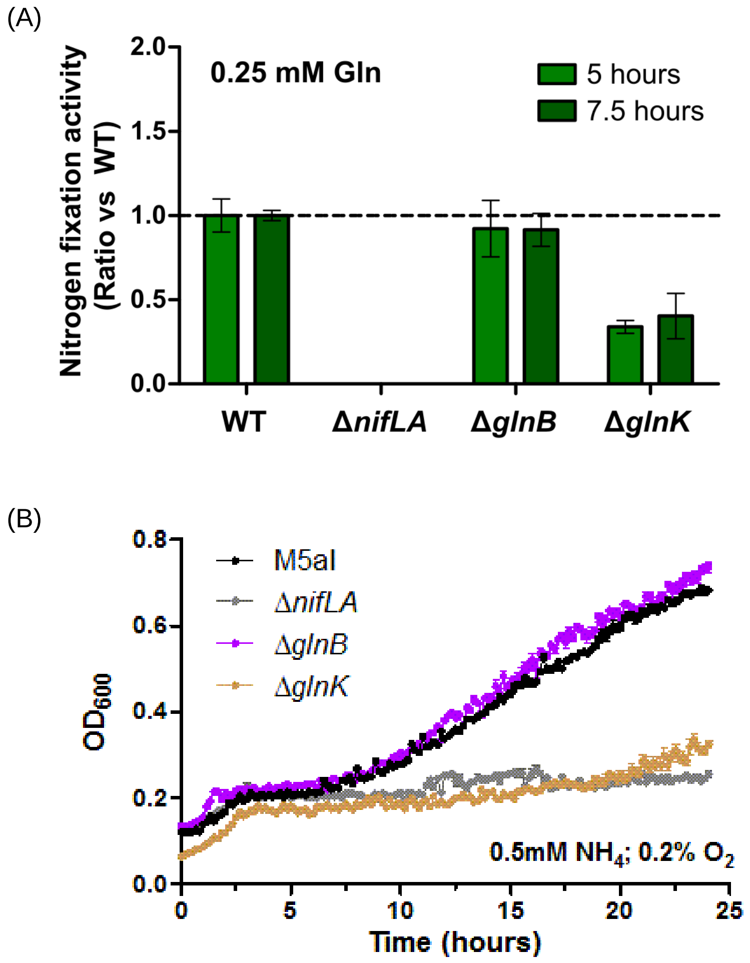

Supplementary Figure 13. **(A)** Effects of Δ*glnB* and Δ*glnK* knockout mutations on diazotrophy in the presence of limiting glutamine. M5a1 wild-type, Δ*nifLA*, Δ*glnB* and Δ*glnK* strains were cultured for 3 hours following run-out of 0.25 mM glutamine in NFDM medium. Fold-change in nitrogen fixation activity with respect to wild-type. Culture headspace was analysed for ethylene evolution via the acetylene reduction assay. Error bars represent SEM of 3 biological replicates. **(B)** Diazotrophic growth phenotypes of Δ*glnB* and Δ*glnK*. M5a1 wild-type, Δ*nifLA*, Δ*glnB* and Δ*glnK* strains were cultured for 24 hours in NFDM medium supplemented with 0.5 mM NH_4Cl and micro-aerobic conditions (0.2% O_2, 1% CO_2 and 98.8% N_2) in 96 well culture plates. OD_{600} was measured at 10-minute intervals. Error bars represent SEM of 3 biological replicates (200ul).
